# The effects of a salsa dance intervention in young people with mild to moderately severe depressive symptoms

**DOI:** 10.1017/S0033291726104991

**Published:** 2026-07-15

**Authors:** Brennan Delattre, Joshua E.J. Buckman, Catherine J. Harmer, Susannah E. Murphy

**Affiliations:** 1 Psychiatry Department, University of Oxford, UK; 2 Oxford Health NHS Foundation Trust, UK; 3 Department of Clinical, Education, and Health Psychology, University College London, UK; 4 UCL University Clinic Camden NHS Talking Therapies for Anxiety and Depression, North London Foundation Trust, St. Pancras Hospital, UK

**Keywords:** dancing, depression, depressive symptoms, intervention, mental health, mood disorders, salsa dancing, social prescribing, young adults, randomized controlled trial, behavioral activation, social anxiety

## Abstract

**Background:**

The prevalence and burden of depression are increasing among young people. Despite this, relatively few treatments specifically target this critical developmental period, and under-addressed mental health difficulties in youth often have lifelong consequences. Social isolation is commonly reported by young adults and is both a risk and maintenance factor for depression. There is a need for accessible, engaging interventions that can reduce depressive symptoms and improve social connectedness.

**Methods:**

Building on evidence of the therapeutic potential of social dance-based activities for depression, this randomized controlled trial evaluated the efficacy of an 8-week salsa dance intervention for young adults (aged 18–24 years) with mild to moderately severe depressive symptoms. 121 participants were randomly assigned to either a salsa intervention or a waitlist control. Participants completed the Patient Health Questionnaire (PHQ-9), a validated measure of depressive symptoms, and additional mental health measures at baseline, during, and after the intervention.

**Results:**

Participants in the salsa dance condition showed a significantly greater reduction in depressive symptoms than the waitlist control, with a −2.45 PHQ-9 point between-group difference, exceeding the criterion for clinical significance. Both groups reported improvements in social anxiety, generalized anxiety, loneliness, and daily happiness, but salsa participants had significantly greater reductions in social anxiety and greater improvements in daily happiness.

**Conclusions:**

These findings support the value of social dance as a novel, accessible intervention for reducing depressive symptoms. Implementing such programs within a suite of wellbeing-oriented interventions for young people could provide cost-effective mental health benefits.

## Introduction

Young adults worldwide are facing the dual problem of increasing prevalence of common mental disorders, such as depression and anxiety, and reduced effectiveness of first-line treatments for these conditions compared to older adults (COVID-19 Mental Disorders Collaborators, 2021; Patel et al., 2023; Saunders et al., 2025; Strawn et al., 2023; Cipriani et al., 2018). These issues are compounded by the fact that depression in young adults (aged 18–25 years old, as defined by the World Health Organization [2024]) remains relatively under-studied. For example, the mean age of participants in the largest meta-analysis of antidepressant efficacy was 44 years (Cipriani et al., 2018), and even in individual-patient data meta-analyses investigating the moderating effect of age on treatment outcomes, the mean age was 42 years (Buckman et al., 2021b). This gap in the evidence base persists despite recognition that the onset of depression peaks between the ages of 15 and 25 (Solmi et al., 2022), and that young adults are less likely to seek treatment (Pettit et al., 2017) and more likely to drop out of treatment once started relative to older adults (Barnett et al., 2022).

There is therefore a need for more effective and readily accessible treatments for depression in young people – particularly interventions that they perceive to be ‘for them’ rather than generic ‘one-size fits all’ approaches that may not align with their particular needs (Barnett et al., 2021). This is critical, as unaddressed or inadequately treated mental health difficulties in youth often persist into adulthood, contributing to long-term individual and societal difficulties (Layard & Clark, 2015). For example, the likelihood of a chronic course and multiple relapses or recurrences is much higher if depression is not prevented or adequately treated in young people (Buckman et al., 2018; Saunders et al., 2021). Young adults, including both university students and those not engaged in education, employment, or training, typically show poorer outcomes from psychological therapies for depression and are more likely to discontinue treatment compared with other age groups (Buckman et al., 2021c). They also report high levels of social isolation and loneliness, as well as difficulties with emotional processing, which are known to be both risk and maintenance factors for depression (Moeller & Seehuus, 2019).

Depression commonly disrupts social functioning and connectedness, which is particularly important for healthy development during adolescence (Jose & Lim, 2014; Viana & Stevens, 2013). A growing body of literature has highlighted the potential social and mental health benefits of social dance and movement (Delattre et al., 2024; Noetel et al., 2024; Lakes et al., 2016; Shuper Engelhard & Vulcan, 2021; Hyvönen, Pylvänäinen, Muotka, & Lappalainen, 2020). However, these effects have not yet been systematically investigated in the context of depression, and thus it remains unclear whether social dance could be an effective primary or adjunctive treatment for depression (Delattre et al., 2024). The impetus for the present study was the idea that learning to dance within a group setting could help to reduce loneliness, foster social connectedness and enjoyment, and promote a sense of achievement in young people. Such experiences may provide the types of behavioral and emotional experiences theorized to be central to recovery in cognitive behavioral and behavioral activation treatments for depression (Beck, Rush, Shaw, & Emery, 1979; Lewinsohn, 1975; Martell, Dimidjian, & Hermann-Dunn, 2010). Additionally, the cost-effectiveness, scalability, and community-based nature of social dance could make it a viable and valuable addition to existing offerings for depression treatment in healthcare settings.

Before undertaking the present study, we conducted a consultation with young adults to explore their interest in a social dance intervention for young people with depression and what form of social dance they might find the most acceptable. Their collective feedback indicated a clear preference for salsa dance, and this was therefore chosen as the intervention for the present study. Accordingly, this study aimed to investigate the potential efficacy of a salsa dance group-based intervention for young people with mild to moderately severe depressive symptoms, compared to a waitlist control, and to investigate putative mechanisms of change. It was hypothesized that participants in the salsa dance condition would experience larger reductions in depressive symptoms compared to those in the waitlist control group.

## Methods

### Patient and participant involvement

To ensure that the social dance and movement activity selected for the study was acceptable and relevant to the target population, young people with and without lived experience of depression and anxiety from an Oxford-based Youth Advisory Group were consulted through a Patient and Participant Involvement (PPI) process (see Supplementary Materials for additional details). They were provided with videos of different social movement activities and asked to imagine what interests and concerns their peers might have. Based on their feedback, salsa dancing was selected as the study intervention activity.

### Participants

Individuals aged 18 to 24 years old at the time of consent who self-reported symptoms of depression were invited to take part in this research. Participants were identified using validated depressive symptom thresholds, reflecting common symptom-based approaches to identifying and treating depression in community, primary care, and youth mental health settings. Those with a Patient Health Questionnaire (PHQ-9) score between 5 and 19 at the time of screening were eligible to participate, so long as they did not self-report a current or recent diagnosis of any psychotic disorder (e.g. bipolar, schizophrenia, schizoaffective disorder), substance use disorder, eating disorder, or personality disorder. Individuals scoring in the severe symptom range on the PHQ-9 (i.e. ≥20) were excluded. Individuals were also not eligible for the study if they self-reported being unfit for light physical exertion, or if they regularly attended partner dance classes/events within the last 6 months. A formal diagnosis of depression, current or past use of antidepressant medication, or current/past engagement in psychological therapies were not inclusion or exclusion criteria.

### Design

This study was a randomized controlled trial with a mixed, two-group design. The intervention group was required to attend at least six out of 8 weeks of salsa classes, while the waitlist group followed the same assessment schedule but received the option to participate in the dance classes after a 12-week delay.

### Outcome measures

#### Primary outcome

The primary outcome in this study was change in depressive symptom severity (as assessed by PHQ-9 score) over time – from baseline (T0) to the study endpoint at 12 weeks post-baseline (T3). Clinically meaningful change was operationalized as the minimum clinically important difference (MCID), defined as the smallest reduction in depressive symptoms that matters to patients, and in this case assigned as a −1.7-point change on the PHQ-9, a figure established via a cohort study of 400 patients in UK primary care by Kounali et al. (2022).

*Power analysis.* An *a priori* power analysis was performed for the primary comparison (change in PHQ-9 score from pre- to post-intervention vs. waitlist control) for a moderate effect size on the PHQ-9 (Kroenke, Spitzer, & Williams, 2001). A power calculation for the sample size necessary to detect a statistically significant difference between the two groups at *p* < 0.05, power = 0.9, and Cohen’s *d* = 0.5 was performed using G* Power 3.1 (Faul, Erdfelder, Buchner, & Lang, 2009). The required sample size was *n* = 91 study completers across both groups. Based on experience recruiting for similar studies, an approximate 35% dropout rate was predicted, and 121 participants were recruited. Analyses were conducted on a per-protocol basis.

#### Secondary outcomes

Changes in depressive symptom severity (PHQ-9) were also measured at T1 (4 weeks) and T2 (8 weeks) post-baseline. In addition, generalized anxiety, social anxiety symptoms, loneliness, social anhedonia, and daily happiness were measured at T0, T1, T2, and T3, respectively (Table 1).

**Table 1.** Standardized questionnaires administered and administration timeline Table 1. long description.

Variable	Assessment tool	References	Scoring description	Administration timeline
Depression	Patient Health Questionnaire–9 (PHQ-9)	Kroenke, Spitzer, & Williams (2001)	The PHQ-9 is comprised of nine items scored from 0 (not at all) to 3 (nearly every day), in which participants indicate their agreement with types and frequencies of depressive symptoms they experienced over the past 2 weeks. PHQ-9 scores represent the total summed score from the nine items, with higher scores indicating greater severity of depression. In terms of clinical score ranges, scores of 5–10 indicate mild depression; scores of 11–15 indicate moderate depression; scores of 16–19 indicate moderately severe depression; and scores of 20 or above indicate severe depression.	**Eligibility** (prior to enrollment); **T0** (baseline); **T1** (intervention midpoint and/or 4 weeks after T0); **T2** (following the last salsa class and/or 8 weeks after T0); **T3** (4 weeks after the last salsa class and/or 12 weeks after T0)
Social anxiety	Social Anxiety Scale for Adolescents (SAS-A)	La Greca & Lopez (1998)	The SAS-A has 18 items pertaining to different aspects of social anxiety scored from 1 (not at all) to 5 (all the time). SAS-A is scored as the total summed score from all of the items, and higher scores indicate more social anxiety.	**T0** (baseline); **T2** (following the last salsa class and/or 8 weeks after T0); **T3** (4 weeks after the last salsa class and/or 12 weeks after T0)
Social anhedonia	Anticipatory and Consummatory Interpersonal Pleasure Scale (ACIPS)	Gooding & Pflum (2014)	The ACIPS is a 17-item Likert scale measure scored from 1 (very false for me) to 6 (very true for me) with statements about pleasure experienced from social interactions. The ACIPS has one reverse-scored item, and the ACIPS score is the summed total of all items; higher scores on the ACIPS correspond to lower social anhedonia.	**T0** (baseline); T1 (intervention midpoint and/or 4 weeks after T0); **T2** (following the last salsa class and/or 8 weeks after T0), **T3** (4 weeks after the last salsa class and/or 12 weeks after T0)
Anxiety	Generalized Anxiety Disorder Assessment (GAD- τ )	Spitzer, Kroenke, Williams, & Löwe (2006)	The GAD–7 has seven items scored from 0 (not at all) to 3 (nearly every day), and participants are asked to indicate which symptoms and at what frequency they have experienced the symptoms over the past 2 weeks. GAD–7 is scored as the summed total of participants’ answers on each item. Higher scores indicate greater anxiety.	**T0** (baseline); **T1** (intervention midpoint and/or 4 weeks after T0); **T2** (following the last salsa class and/or 8 weeks after T0); **T3** (4 weeks after the last salsa class and/or 12 weeks after T0)
Loneliness	UCLA 3-Item Loneliness Scale (UCLA-L-3)	ONS (2018)	The short form UCLA-L for young people was chosen due to the use of simplified language and was used to assess subjective feelings of loneliness and social isolation. The scale consists of three items, such as ‘How often do you feel left out?’, scored from 1 (hardly ever or never) to 3 (often). Scores are summed for this item, and higher scores reflect greater feelings of loneliness.	**T0** (baseline), **T1** (intervention midpoint and/or 4 weeks after T0); **T2** (following the last salsa class and/or 8 weeks after T0); **T3** (4 weeks after the last salsa class and/or 12 weeks after T0)
	Direct 1-ltem Loneliness Question (UCLA-L-1)	Badcock & Lim (2021)	The direct loneliness question, how often do you feel lonely, is scored from 1 (never) to 5 (often/always) and was used in addition to the UCLA-L questions following guidance from the creator of the assessment tool. Higher scores indicate more loneliness.	**T0** (baseline); **T1** (intervention midpoint and/or 4 weeks after T0); **T2** (following the last salsa class and/or 8 weeks after T0); **T3** (4 weeks after the last salsa class and/or 12 weeks after T0)
Empathy	Basic Empathy Scale (BES)	Jolliffe & Farrington (2006)	The BES has 20 items encompassing measures of both cognitive and affective empathy, scored from 1 (strongly disagree) to 5 (strongly agree). The BES has reverse-scored items and is scored as the summed total of the composite scale; higher scores indicate greater empathy.	**T0** (baseline), **T2** (following the last salsa class and/or eight weeks after T0)
Daily happiness	Ecological Momentary Assessment (EMA)	Pirla, Taquet, & Quoidbach (2023)	Participants completed daily happiness snapshot measures using ecological momentary assessment; they received a text message from the study phone once per day over the course of the study at varied times (random sampling) with a one-item question: ‘How happy do you feel right now? (Please respond with a number 1–10, where 1 indicates very unhappy and 10 indicates very happy.)’*	**Daily**, from 1 day after **T0** until **T3**

#### Exploratory measures

*Class experiences.* In order to examine whether participants’ experiences of social dance moderated any improvements to their mood, intervention participants completed a short online survey consisting of three Likert-scale questions via their smartphone (or a device from a member of the research team) directly following each dance class, prior to leaving class. These questions were: 1) How much did you enjoy today’s class? 2) How connected do you feel? and 3) How happy do you feel? Scores on these three items were averaged to give a single combined score.

*Tasks.* Social and emotional processing and social interaction tasks were included as exploratory outcome measures to investigate potential mechanisms of change. These tasks were completed online at T0 (baseline) and T2 (8 weeks later). A full list of tasks and outcomes is available in the Supplementary Materials.

*Acceptability of intervention.* Following the last study timepoint, intervention participants completed questions regarding the acceptability of the intervention (see Supplementary Materials).

#### Ethical approval

Ethical approval was obtained from the Central University Research Ethics Committee (CUREC Reference: R85689/RE001). The study was preregistered on ClinicalTrials.gov (ID: NCT05963581). While social dance classes were conducted in person, all data were collected online (or, in the case of the daily mood measures, by text message).

### Procedure

Participants were recruited from in and around Oxfordshire, United Kingdom. After reviewing the online participant information sheet and completing preliminary screening (PHQ-9), eligible individuals were contacted by a research team for a phone-based eligibility assessment. Basic demographic information (age, gender, ethnicity) and mental-health specific information (whether participants had seen a health professional regarding their mental health, were taking medication for mental health, and had a diagnosis of depression and/or anxiety) were collected. All participants gave informed consent. Once enrolled, participants were randomly assigned to the experimental or control condition according to a pregenerated Sealed Envelope Ltd (2022) list prepared by a member of the research team and stratified by gender.

In the experimental condition, participants completed 8 weeks of a salsa dancing course in Oxford. Salsa classes were hosted by Oxford-based salsa instructors associated with local dance societies (Oxford University Salsa Society, Linacre College Latin Dance Society, and Teddy Hall Latin Dance Society). Salsa classes were taught by six instructors (three male, three female) of varied nationalities (UK, US, Italy, Romania, Spain) in different combinations. A member of the research team was in attendance at all classes for observation. Salsa classes took place in central Oxford, and participants were able to enter the salsa classes the same way as society members who were not participating in the research, ensuring research participants were indistinguishable from non-study attendees.

Participants assigned to the waitlist **control condition** completed questionnaires on the same schedule as individuals in the experimental condition and received the same compensation for participating in the study (£100). These participants were offered the opportunity to complete the 8-week salsa course after a 12-week waiting period.

## Results

### Descriptive statistics

**Figure 1.**
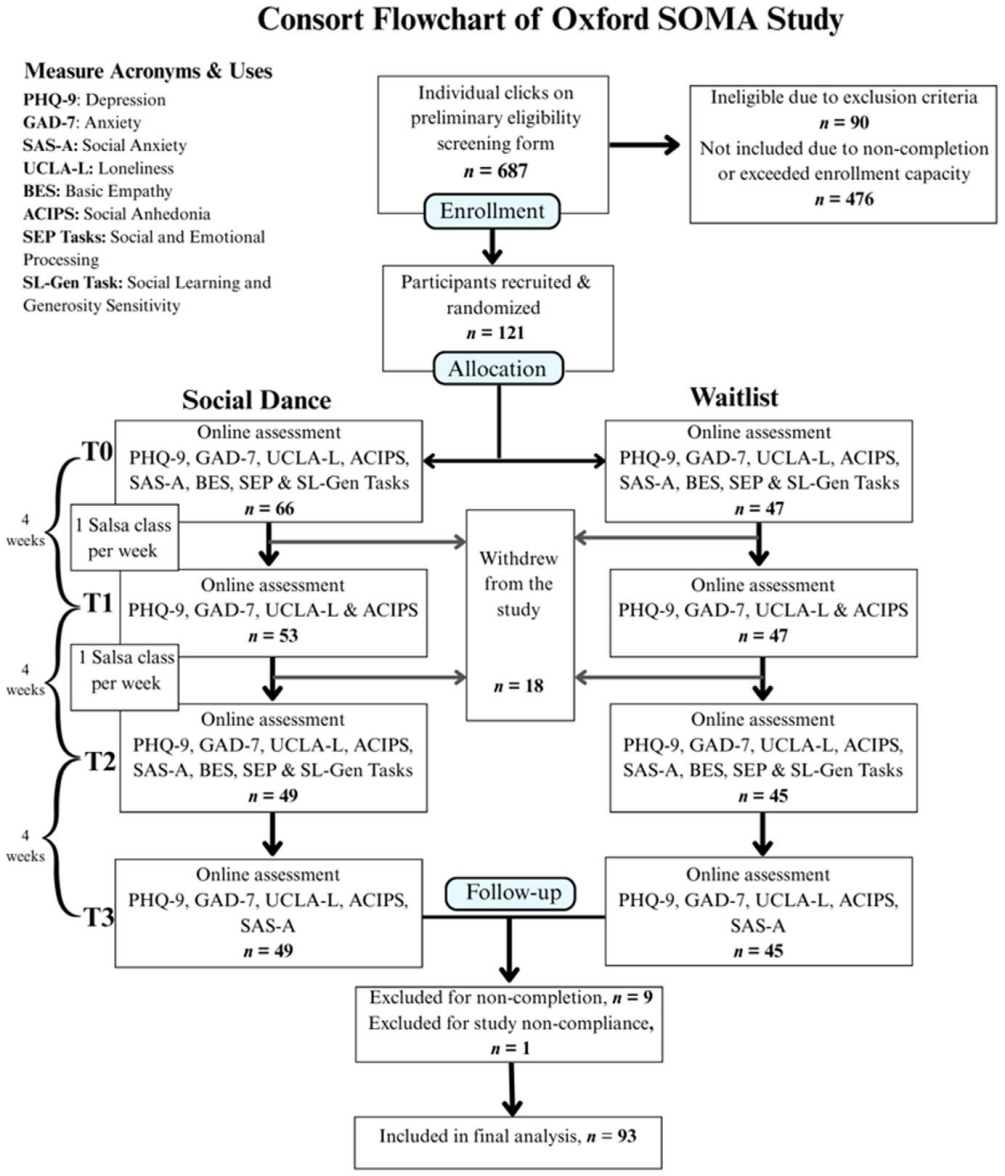
Consort flowchart. Figure 1. long description.

121 participants were enrolled in this study over the course of three waves of data collection (summer, autumn, winter) between July 2023 and February 2024. Forty-nine experimental condition participants and 45 control condition participants completed the study (77.7%) (Figure 1; for dropout reasons provided, see Supplementary Materials). The groups were well-matched on demographic and clinical characteristics. One participant was withdrawn after data collection for study noncompliance, yielding an analyzed sample of 93 participants (19 men, 73 women, 1 nonbinary individual; aged between 18 and 24, *M* = 21.46, *SD* = 1.67); see Table 2 for demographics.

**Table 2.** Demographics of included participants Table 2. long description.

Variable	Social dance (*n* = 49)	Waitlist (*n* = 44)
Gender, *n* (%)		
Female	38 (77.6%)	35 (79.5%)
Male	10 (20.4%)	9 (20.5%)
Nonbinary	1 (2.0%)	0 (0.0%)
Age, mean (SD)	21.4 (1.7)	21.5 (1.6)
Ethnicity, *n* (%)		
White British or White other	34 (69.4%)	28 (63.6%)
Black British or Black other	2 (4.1%)	2 (4.5%)
Asian British or Asian other	10 (20.4%)	10 (22.7%)
Mixed ethnicities	3 (6.1%)	4 (9.1%)
Education, *n* (%)		
Secondary school	2 (4.1%)	1 (2.3%)
A-levels	20 (40.8%)	21 (47.7%)
Undergraduate	23 (46.9%)	17 (38.6%)
Masters	4 (8.2%)	5 (11.4%)
Mental health-related medication (currently), *n* (%)		
Yes	7 (14.3%)	8 (18.2%)
No	41 (83.7%)	36 (81.8%)
Antidepressant medication (other purpose)	1 (2.0%)	0 (0.0%)
Seen a professional for mental health, *n* (%)		
Yes: currently	2 (4.1%)	2 (4.5%)
Yes: in the past	25 (51.0%)	23 (52.3%)
Yes: both currently and in the past	1 (2.0%)	2 (4.5%)
Yes: timeline unspecified	6 (12.2%)	5 (11.4%)
No	15 (30.6%)	12 (27.3%)
Diagnosis of depression/anxiety, *n* (%)		
Depression	2 (4.1%)	2 (4.5%)
Anxiety	4 (8.2%)	4 (9.1%)
Both depression and anxiety	8 (16.3%)	9 (20.5%)
Self-diagnosis or unsure	4 (8.2%)	4 (9.1%)
No diagnosis	31 (63.3%)	25 (56.8%)
Baseline measures		
PHQ-9, mean (SD)	9.33 (3.88)	9.38 (3.80)
SAS-A, mean (SD)	47.12 (13.46)	51.89 (14.89)
ACIPS, mean (SD)	82.18 (8.37)	83.48 (11.21)
GAD-7, mean (SD)	8.20 (3.94)	8.27 (4.28)
UCLA-3, mean (SD)	6.02 (1.36)	6.20 (1.76)
UCLA-1, mean (SD)	3.73 (0.84)	3.98 (0.95)
BES, mean (SD)	62.24 (4.14)	60.47 (4.54)

### Intervention effects

#### Primary outcome: Depressive symptom severity

**Figure 2.**
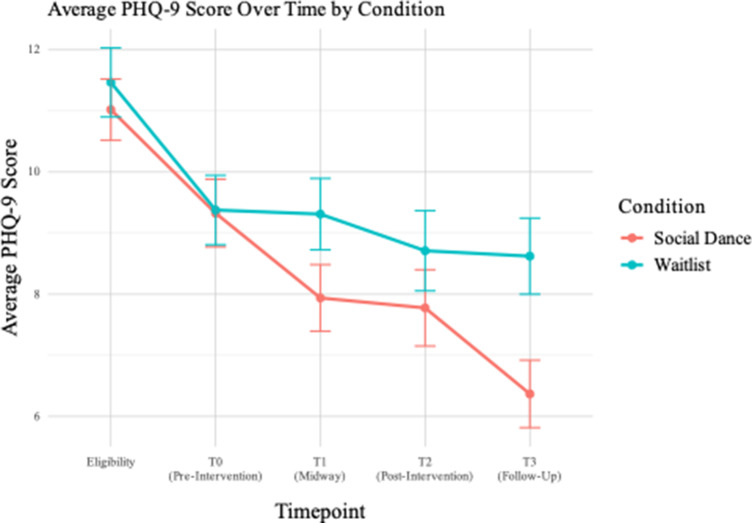
Average PHQ-9 score over time by condition. *Note:* The eligibility timepoint is not reported in the above analyses, which focus on pre-post intervention symptom change (from T0 to T3). *n = 93.* Figure 2. long description.

A linear mixed-effects model (LMM) was fitted to examine the effect of condition (social dance vs. waitlist) on PHQ-9 scores over the four timepoints (T0, T1, T2, and T3; Figure 2), with participants included as a random intercept in the model to account for repeated measures. Baseline PHQ-9 scores did not significantly differ between the social dance and waitlist groups (*B* = 1.15, *p* = .632). There was evidence of a main effect of time, *B* = −3.43, at T3 relative to baseline (SE = 0.38, *t*(364) = −9.04, *p* < .001), suggesting that PHQ-9 scores decreased over time in both conditions. As predicted, there was evidence of a condition-by-time interaction, *B* = 1.56, SE = 0.55, *t*(364) = 2.82, *p* = .005, suggesting that social dance participants experienced a significantly greater reduction in PHQ-9 score compared to those in the waitlist condition. Standardized estimates showed a moderate-sized interaction effect (β = 0.38, 95% CI[0.11,0.64]). At T3, participants in the social dance group had significantly lower PHQ-9 scores (*M =* 6.37, *SD* = 3.87) compared to the waitlist group (*M =* 8.82, *SD* = 4.01). This corresponds to a between-group difference in change scores of approximately 2.45 PHQ-9 points, with a large between-group effect size (Cohen’s d = −0.92, 95% CI[−1.53,-0.32]); see Table 3. There was also a large within-group reduction in PHQ-9 scores for the social dance group from T0 to T3 (*d* = 1.11, 95% CI[0.71,1.52]), while the waitlist group showed a small, nonsignificant change over the same period (*d* = 0.29, 95% CI[−0.13,0.71]).

**Table 3.** Mean and standard deviation of PHQ-9 scores by group and timepoint Table 3. long description.

	Eligibility	T0	T1	T2	T3
Social dance	11.02 ± 3.51	9.33 ± 3.88	7.94 ± 3.82	7.78 ± 4.37	6.37 ± 3.87
Waitlist	11.41 ± 3.82	9.59 ± 3.57	9.50 ± 3.75	8.84 ± 4.36	8.82 ± 4.01

#### Secondary outcomes

**Figure 3.**
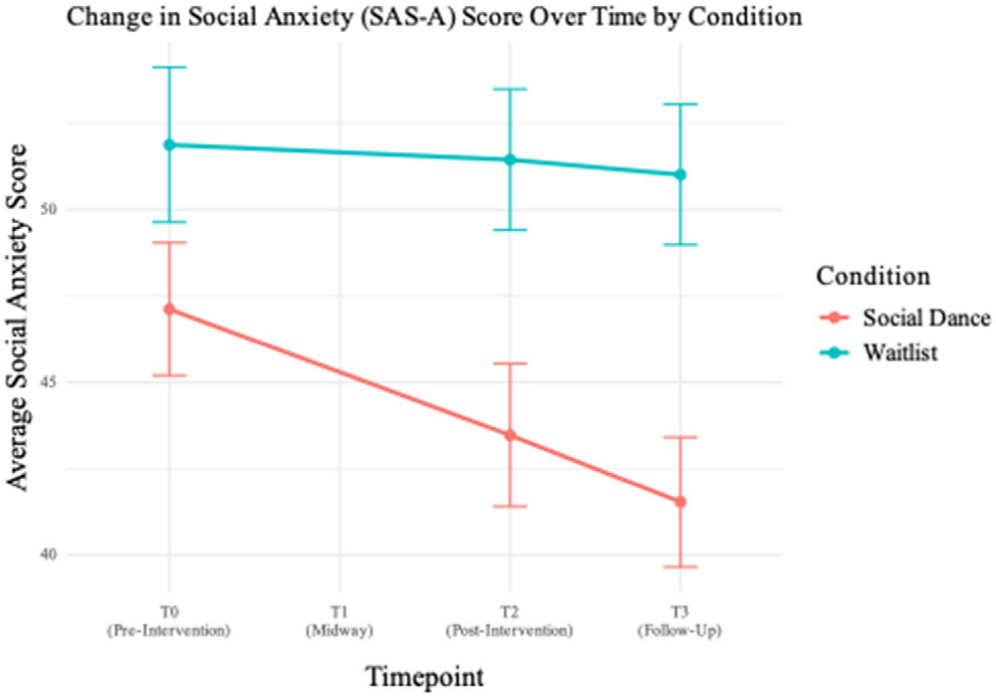
Change in SAS-A scores over time by condition. *Note:* this measure was only administered at baseline (T0), following the salsa classes (T2), and follow-up (T3), e.g. not administered at T1. *n = 93.* Figure 3. long description.

*Social anxiety (SAS-A Score).* An LMM was fitted to examine the effects of condition and timepoint on SAS-A score, with participants included as a random intercept. Importantly, there was a significant condition by timepoint interaction (*B* = 4.73, SE = 1.93, *t*(182) = 2.45, *p* = .015), indicating that participants in the social dance condition experienced a greater reduction in social anxiety scores from T0 to T3 compared to those in the waitlist condition (Figure 3). The standardized effect for this interaction was moderate (*β* = 0.33, 95% CI [0.06,0.60]). At T3, the between-group effect size was large (Cohen’s *d* = −1.44, 95% CI[−2.32,–0.57]), with the social dance group experiencing greater improvements. Within-group effect sizes further supported the differential improvement; in the salsa group, social anxiety scores dropped significantly from T0 to T2 (*d* = 0.56, 95% CI[0.15,0.96]) and from T0 to T3 (*d* = 0.85, 95% CI[0.44,1.26]). By contrast, the waitlist group experienced negligible change across timepoints (T0 to T3: *d* = 0.13, 95% CI[−0.29,0.55]).

Baseline social anxiety varied widely between participants (random intercept variance = 148.60, *SD* = 12.19). Mean baseline scores were similar across groups (*B* = 4.76, SE = 2.88, *t*(124.14) = 1.66, *p* = .10).

*Social anhedonia (ACIPS Score).* Social anhedonia increased in the waitlist but not in the social dance condition, reflecting a significant condition by time interaction (β =−0.42, 95% CI[−0.70,–0.15]). At T3, the waitlist group had lower ACIPS scores than the salsa group (*t*(273) = −3.03, *p* = .003; *d* = 0.64, 95% CI[−0.23, 1.51]). No significant interactions emerged at earlier timepoints. Within the waitlist group, ACIPS scores decreased significantly from T0 to T3 (*d* = 0.59, 95% CI[0.17, 1.02]), indicating increased social anhedonia over time. In light of this, a mediation analysis was conducted to examine whether the positive impact of social dance on depressive symptoms was mediated by ACIPS. The average causal mediation effect (ACME) was not significant (ACME = 0.02, 95% CI[−0.28, 0.36], *p* = .839), indicating no evidence of mediation (neither full nor partial). Less than 1% of the total effect was mediated by ACIPS.

*Generalized anxiety (GAD) and loneliness (UCLA-L).* Both generalized anxiety and loneliness decreased over the study period (generalized anxiety: *B* = −2.65, SE = 0.58, *t*(273) = −4.55, *p* < .001; three-item loneliness: *B* = −1.22, SE = 0.20, *t* = −6.04, *p* < .001; direct loneliness item: *B* = −0.51, SE = 0.14, *t* = −3.56, *p* < .001); however, there was no significant main effect of condition (anxiety: *B* = 0.07, SE = 0.91, t(186) = 0.08, *p* = .94; three-item loneliness: *B* = 0.18, SE = 0.33, *t* = 0.55, *p* = .58; direct loneliness item: *B* = 0.24, SE = 0.20, *t* = 1.18, *p* = .24) or condition by time interaction (anxiety: *B* = 1.31, SE = 0.85, *t*(273) = 1.55, *p* = .12; three-item loneliness: *B* = 0.47, SE = 0.30, *t* = 1.60, *p* = .110), suggesting that the social dance participants did not experience different reductions in anxiety or loneliness compared with the waitlist group (see Supplementary Materials). Additionally, random effects showed substantial baseline variability in anxiety (variance = 10.8, *SD* = 3.29), suggesting that individual differences may have obscured potential intervention effects on anxiety.

**Figure 4.**
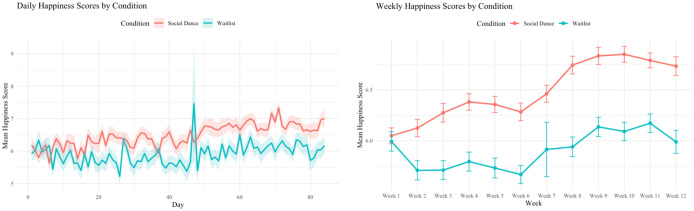
Daily and weekly happiness scores by condition (*n* = 49). *Note:* On the daily happiness plot, shaded areas indicate ± standard error. Figure 4. long description.

*Daily happiness.* Daily happiness scores were collected from the day after participants completed their T0 assessment until the day they completed their T3 assessment. Data were double-entered for ~44% of records, yielding 98.5% agreement between scores (3523 matching out of 3577 total scores), indicating excellent transfer reliability. To standardize observation windows, data were truncated at Day 84 (end of week 12) (Figure 4).

Participants’ raw daily happy mood scores and their averaged weekly happiness scores (from Weeks 1–12) were analyzed by condition. An LMM was fitted to examine the effect of day, condition (salsa vs. waitlist), and their interaction on daily mood ratings. The model included a random intercept for participants (*n* = 93) and was fit using Restricted/Residual Maximum Likelihood (REML). There was a significant day-by-condition interaction, *b* = −0.0053, SE = 0.0017, *t*(6567) = −3.20, *p* = .0014, indicating that the slope of mood change over days was significantly less steep for the waitlist condition compared to the social dance condition. The week-by-condition interaction was also significant, *b* = −0.037, SE = 0.012, *t*(6567) = −3.12, *p* = .0018, indicating a slower increase in weekly happiness averages for the waitlist group compared to the experimental group. Sensitivity analyses suggested that these results were robust to missing data (see Supplementary Materials).

#### Exploration of potential mechanisms

*Empathy (BES).* Empathy did not moderate treatment effects; there was no evidence of moderation in the two-way or three-way interactions of empathy, condition, and time (*B* = 0.08, SE = 0.17, *t*(270) = 0.45, *p* = .65).

*Class experiences.* Self-reported positive class experience significantly moderated changes in depressive symptoms within the social dance group (*B* = 1.78, SE = 0.42, *t*(188) = 4.28, *p* < .001; see Supplementary Materials). Specifically, individuals reporting more positive class experience had significantly lower PHQ-9 scores at various timepoints compared to those reporting less positive class experience.

*Task data.* Across exploratory social/emotional processing tasks (see Supplementary Materials), there were no significant condition-by-time interactions. This held (a) in the full sample, and (b) when restricting analysis to individuals with PHQ-9 scores over 10 at baseline (*n* = 45) and (c) when excluding those who reported taking a medication with antidepressant effects (*n* = 17), given known antidepressant medication effects on emotional processing.

#### PHQ-9 by *baseline* severity

Given the wide range of participants’ PHQ-9 scores (0–19) at baseline (T0), and as baseline PHQ-9 severity largely impacts endpoint scores (Buckman et al., 2021a), we conducted an exploratory analysis to look at the effect of baseline PHQ-9 severity on individuals’ depressive symptom trajectories over time (see Supplementary Materials). There was a significant timepoint × T0 severity interaction (*B* = 2.96, SE = 0.75, *t*(364) = 3.96, *p* < .001), indicating that baseline depression severity influenced symptom change over time. The main effect of T0 severity was also significant (*B* = −3.82, *p* < .001), suggesting that participants with lower baseline symptoms consistently reported lower PHQ-9 scores throughout the study. However, the three-way interaction between condition, timepoint, and T0 severity was not significant (*B* = −1.52, SE = 1.08, *t*(360) = 1.41, *p* = .16), indicating that the effect of the salsa intervention did not significantly differ by baseline depression severity. In other words, while baseline PHQ-9 scores were associated with the amount of overall improvement, participation in the social dance intervention was associated with greater symptom improvement regardless of baseline symptom severity.

*Acceptability.* Salsa participants completed quantitative and qualitative acceptability of intervention measures at T3. Participants responded to 10 Likert-scale acceptability questions, as well as two free-response questions. Overall, acceptability responses were positive; for histograms of responses to each quantitative item, as well as complete free responses, please see Supplementary Materials. Notably, when responding via Likert scale to ‘How acceptable were the salsa classes as part of this research to you?’, the majority of participants selected ‘completely acceptable’, followed by ‘acceptable’, followed by ‘no opinion’ (a minority of participants); no participants reported that the intervention was unacceptable or completely unacceptable. Free text themes included: the value of enthusiastic, friendly and welcoming instructors; the influence of the experience level of other people in the class; motivation to attend; mood improvements after class (with variable durations); a preference for consistent attendees in the classes, giving the opportunity to build trust with other class participants; suggestions for smaller class sizes; occasional discomfort dancing with strangers or individuals of a specific gender; and a preference in some cases to learn a single role (leading or following) initially. Several participants noted other factors that influenced their mental health over the course of the longitudinal intervention (e.g. university term, positive and negative external life events) and made a point to say that they were not sure that the mental health measures accurately reflected this and captured the impact of the intervention in light of their broader context and mental health landscapes.

## Discussion

### Intervention effects

In this randomized controlled trial, participants in the social dance condition experienced a meaningful reduction in depression symptoms relative to those in the waitlist control condition. Specifically, intervention participants experienced an average within-group reduction of −3.0 points on the PHQ-9, and the between-group difference at the final timepoint was 2.45 points, exceeding the threshold for the MCID. This corresponds to a large standardized effect size (Cohen’s d = −0.92); larger standardized effect sizes are expected in comparisons against waitlist or inactive control conditions, and this context should be considered when interpreting the magnitude of the observed effect. The within-group reduction in PHQ-9 scores for the intervention group (but not the control group) exceeded the NICE criterion for clinically meaningful change (Pilling et al., 2011). As expected, baseline depression severity impacted individuals’ depression trajectories over time – participants starting with higher PHQ-9 scores remained higher over the course of the study – but irrespective of initial severity, participating in social dance was associated with greater depression symptom improvement compared to the waitlist condition both in those with baseline depression indicative of a probable major depressive episode and in those with sub-threshold symptoms.

The intervention was associated with improvements across multiple secondary outcomes. While both groups experienced improvements over time in social anxiety, generalized anxiety, loneliness, and daily happiness, the intervention group participants experienced significantly greater reductions in social anxiety and a steeper increase in daily happiness than the waitlist group participants.

In terms of the exploratory outcomes, there was some evidence that the waitlist condition participants experienced an increase in social anhedonia over time, and this was not seen in the social dance participants. This may be because social dance helps to preserve or enhance interpersonal enjoyment, which might otherwise diminish over time in individuals with untreated depression in a waitlist context. Although social anhedonia did not mediate the effect of social dance on depression in this sample, there was a large variation of baseline social anhedonia across participants, which may have limited the sensitivity to detect an effect. Future research should further examine the impact of social dance on social anhedonia. If social dance acts by increasing interpersonal pleasure, this mechanism could also help to explain the absence of significant condition × time interactions on social and emotional processing tasks; the active ingredients of social dance may not correspond to the cognitive-emotional constructs typically sensitive to pharmacological interventions (Harmer, Duman, & Cowen, 2017).

Overall, social dance was also found by participants to be acceptable in both qualitative and quantitative self-report acceptability measures.

### Limitations

A key challenge in this study and the social dance literature at large is selection bias. Those who opt in to studies like this are likely to already believe that dance interventions will be helpful and enjoyable (Delattre et al., 2024). However, this is unlikely to have been different across randomized conditions, and expectancy is known to be a moderator in other treatments for depression (Constantino et al., 2011; Delgadillo, Moreea, & Lutz, 2016). Further, increasing activities that give one a sense of enjoyment is theorized to be a moderator of change in depression and is therefore a key feature of evidence-based psychological therapies for depression (Beck, Rush, Shaw, & Emery, 1979; Martell, Dimidjian, & Hermann-Dunn, 2010). As such, there is value in studying the efficacy of an intervention for the subset of the population that would be most likely to benefit from it and choose it as a treatment option. Further selection biases may have arisen due to the setting and locations for recruitment. However, efforts were made to ensure study advertisements were both gender and ethnically neutral by using a cartoon illustration of two brightly colored dancing figures (see Supplementary Materials), by advertising in both university and town locations, and by holding classes on weekday evenings, making them accessible to those working during weekdays. These decisions were made following consultation with the PPI codesign participants with the aim of increasing the potential for real-world utility.

Having a waitlist control condition could inflate relative effects between the intervention and control conditions as intervention participants receive additional attention, social interaction, and expectancy benefits, while those in the waitlist condition could experience demoralization or reduced motivation (Cunningham, Kypri, & McCambridge, 2013; Furukawa et al., 2014). In such designs, comparisons of an active intervention against a waitlist or inactive control often yield larger standardized effect sizes than comparisons against active or placebo controls, and this context is important when interpreting the magnitude of observed effects. Indeed, while large standardized effect sizes were observed in the present study, these should be interpreted with caution, particularly where outcome variability may be restricted due to the PHQ-9 inclusion range (5–19), the relatively homogenous young adult sample, and use of a waitlist control group. At the same time, larger effects are not uncommon in trials comparing active interventions to inactive controls (Furukawa et al., 2014) and should not be considered implausible in this context. To support transparent interpretation, raw PHQ-9 means and standard deviations are reported alongside between-group differences in change scores expressed in clinically interpretable units. Future studies of this nature should include active control conditions.

Regarding attrition, potential intervention effects in those who did not choose to continue the study could not be examined, and analyses were conducted only with participants who had data at all timepoints (on a per-protocol basis). Missing data mechanisms were not formally assessed, and although attrition was modest and largely due to logistical factors (Supplementary Appendix B), the possibility of bias due to non-random missingness cannot be fully excluded. Additionally, while the study’s primary and secondary outcomes were preregistered, model specifications were not included in the preregistration. The primary PHQ-9 analysis and secondary outcomes were prespecified and confirmatory, while class experiences as a moderator, task-based measures, and acceptability of intervention data were exploratory. Accordingly, findings beyond the primary outcome should be interpreted cautiously and viewed as preliminary pending replication, particularly as no formal adjustment for multiple testing was applied to secondary and exploratory analyses.

The logistical necessities and design of the study did not allow the researcher who collected the data to be blind to the conditions of participants. However, data quality checks and exclusion decisions were completed by other members of the research team who were unaware of group allocation to mitigate any potential bias. Regarding analysis, the study was powered for the main effect of the primary outcome, but possibly underpowered for other analyses, especially mediation and interaction effects; future research should include larger sample sizes to better examine secondary and exploratory measures.

Additionally, this study was not conducted with a clinical sample, even though some participants met criteria for a major depressive episode; future research with clinical populations should be conducted. Accordingly, the present findings should be interpreted as evidence for reductions in depressive symptoms, rather than as evidence of treatment efficacy in a population diagnosed via structured clinical interviews. However, the use of validated symptom thresholds reflects common symptom-based approaches to identifying and treating depression in community, primary care, and youth mental health settings, supporting the real-world relevance of the findings.

Lastly, as with many studies of depression and of dance interventions, the sample was predominantly female; it could be that other forms of social movement activities could reach a wider audience with similar benefits for depressive symptomology.

### Conclusions

The findings of this study provide one of the first rigorous demonstrations that social dance can improve depression and related symptomology in young people. Given its scalability, low cost, and strong acceptability, social dance merits further investigation in clinical settings (such as the NHS) and as part of wellbeing programs in university and community settings.

## Supporting information

10.1017/S0033291726104991.sm001 Delattre et al. supplementary material Delattre et al. supplementary material

## Supplementary material

The supplementary material for this article can be found at http://doi.org/10.1017/S0033291726104991.

## Long descriptions

### Table 1. Long description

The table contains five columns: Variable, Assessment tool, References, Scoring description, and Administration timeline.

* Depression: Measured by P H Q 9 (Kroenke et al., 2001). Nine items scored 0 to 3. Higher scores indicate greater severity (5 to 10 mild, 11 to 15 moderate, 16 to 19 moderately severe, 20 plus severe). Administered at Eligibility, T 0 (baseline), T 1 (4 weeks), T 2 (8 weeks), and T 3 (12 weeks).

* Social anxiety: Measured by S A S A (La Greca and Lopez, 1998). 18 items scored 1 to 5. Higher scores indicate more anxiety. Administered at T 0, T 2, and T 3.

* Social anhedonia: Measured by A C I P S (Gooding and Pflum, 2014). 17 items scored 1 to 6. Higher scores correspond to lower social anhedonia. Administered at T 0, T 1, T 2, and T 3.

* Anxiety: Measured by G A D 7 (Spitzer et al., 2006). Seven items scored 0 to 3. Higher scores indicate greater anxiety. Administered at T 0, T 1, T 2, and T 3.

* Loneliness: Measured by U C L A L 3 (O N S, 2018). Three items scored 1 to 3. Higher scores reflect greater loneliness. Administered at T 0, T 1, T 2, and T 3.

* Loneliness (Direct): Measured by U C L A L 1 (Badcock and Lim, 2021). One item scored 1 to 5. Higher scores indicate more loneliness. Administered at T 0, T 1, T 2, and T 3.

* Empathy: Measured by B E S (Jolliffe and Farrington, 2006). 20 items scored 1 to 5. Higher scores indicate greater empathy. Administered at T 0 and T 2.

* Daily happiness: Measured by E M A (Pirla et al., 2023). One item scored 1 to 10. Administered daily from T 0 until T 3.

Navigate back to Table 1..

### Figure 1. Long description

The flowchart begins at the top with Enrollment. 687 individual clicks on the preliminary eligibility screening form led to 90 exclusions for criteria and 476 not included due to non-completion or capacity. 121 participants were recruited and randomized.

Following Allocation, the study splits into two columns: Social Dance (left) and Waitlist (right).

At T 0 (Baseline):

* Social Dance: n = 66. Online assessment includes P H Q-9, G A D-7, U C L A-L, A C I P S, S A S-A, B E S, S E P and S L-Gen Tasks.

* Waitlist: n = 47. Same assessment battery.

Between T 0 and T 1 (4 weeks, 1 Salsa class per week for the dance group):

* Social Dance: n = 53.

* Waitlist: n = 47.

* Assessment: P H Q-9, G A D-7, U C L A-L and A C I P S.

Between T 1 and T 2 (4 weeks, 1 Salsa class per week for the dance group):

* Social Dance: n = 49.

* Waitlist: n = 45.

* Assessment: Full battery as at T 0.

At T 3 (4 weeks after T 2):

* Social Dance: n = 49.

* Waitlist: n = 45.

* Assessment: P H Q-9, G A D-7, U C L A-L, A C I P S, S A S-A.

A central box indicates 18 participants total withdrew from the study during the T 0 to T 2 period.

At the bottom, under Follow-up, 9 participants were excluded for non-completion and 1 for study non-compliance. The final box states 93 participants were included in the final analysis.

A legend in the top-left defines measure initialisms: P H Q-9 (Depression), G A D-7 (Anxiety), S A S-A (Social Anxiety), U C L A-L (Loneliness), B E S (Basic Empathy), A C I P S (Social Anhedonia), S E P Tasks (Social and Emotional Processing), and S L-Gen Task (Social Learning and Generosity Sensitivity).

Navigate back to Figure 1..

### Table 2. Long description

The table consists of three columns: Variable, Social dance (n = 49), and Waitlist (n = 44).

Gender, n (%):

- Female: 38 (77.6%) in Social dance; 35 (79.5%) in Waitlist.

- Male: 10 (20.4%) in Social dance; 9 (20.5%) in Waitlist.

- Nonbinary: 1 (2.0%) in Social dance; 0 (0.0%) in Waitlist.

Age, mean (S D):

- Social dance: 21.4 (1.7); Waitlist: 21.5 (1.6).

Ethnicity, n (%):

- White British or White other: 34 (69.4%) in Social dance; 28 (63.6%) in Waitlist.

- Black British or Black other: 2 (4.1%) in Social dance; 2 (4.5%) in Waitlist.

- Asian British or Asian other: 10 (20.4%) in Social dance; 10 (22.7%) in Waitlist.

- Mixed ethnicities: 3 (6.1%) in Social dance; 4 (9.1%) in Waitlist.

Education, n (%):

- Secondary school: 2 (4.1%) in Social dance; 1 (2.3%) in Waitlist.

- A-levels: 20 (40.8%) in Social dance; 21 (47.7%) in Waitlist.

- Undergraduate: 23 (46.9%) in Social dance; 17 (38.6%) in Waitlist.

- Masters: 4 (8.2%) in Social dance; 5 (11.4%) in Waitlist.

Mental health-related medication (currently), n (%):

- Yes: 7 (14.3%) in Social dance; 8 (18.2%) in Waitlist.

- No: 41 (83.7%) in Social dance; 36 (81.8%) in Waitlist.

- Antidepressant medication (other purpose): 1 (2.0%) in Social dance; 0 (0.0%) in Waitlist.

Seen a professional for mental health, n (%):

- Yes: currently: 2 (4.1%) in Social dance; 2 (4.5%) in Waitlist.

- Yes: in the past: 25 (51.0%) in Social dance; 23 (52.3%) in Waitlist.

- Yes: both currently and in the past: 1 (2.0%) in Social dance; 2 (4.5%) in Waitlist.

- Yes: timeline unspecified: 6 (12.2%) in Social dance; 5 (11.4%) in Waitlist.

- No: 15 (30.6%) in Social dance; 12 (27.3%) in Waitlist.

Diagnosis of depression/anxiety, n (%):

- Depression: 2 (4.1%) in Social dance; 2 (4.5%) in Waitlist.

- Anxiety: 4 (8.2%) in Social dance; 4 (9.1%) in Waitlist.

- Both depression and anxiety: 8 (16.3%) in Social dance; 9 (20.5%) in Waitlist.

- Self-diagnosis or unsure: 4 (8.2%) in Social dance; 4 (9.1%) in Waitlist.

- No diagnosis: 31 (63.3%) in Social dance; 25 (56.8%) in Waitlist.

Baseline measures, mean (S D):

- P H Q - 9: 9.33 (3.88) in Social dance; 9.38 (3.80) in Waitlist.

- S A S - A: 47.12 (13.46) in Social dance; 51.89 (14.89) in Waitlist.

- A C I P S: 82.18 (8.37) in Social dance; 83.48 (11.21) in Waitlist.

- G A D - 7: 8.20 (3.94) in Social dance; 8.27 (4.28) in Waitlist.

- U C L A - 3: 6.02 (1.36) in Social dance; 6.20 (1.76) in Waitlist.

- U C L A - 1: 3.73 (0.84) in Social dance; 3.98 (0.95) in Waitlist.

- B E S: 62.24 (4.14) in Social dance; 60.47 (4.54) in Waitlist.

Navigate back to Table 2..

### Table 3. Long description

The table contains two rows of data representing the Social dance group and the Waitlist group. The columns represent five timepoints: Eligibility, T 0, T 1, T 2, and T 3.

* Social dance group scores: Eligibility is 11.02 plus or minus 3.51. T 0 is 9.33 plus or minus 3.88. T 1 is 7.94 plus or minus 3.82. T 2 is 7.78 plus or minus 4.37. T 3 is 6.37 plus or minus 3.87.

* Waitlist group scores: Eligibility is 11.41 plus or minus 3.82. T 0 is 9.59 plus or minus 3.57. T 1 is 9.50 plus or minus 3.75. T 2 is 8.84 plus or minus 4.36. T 3 is 8.82 plus or minus 4.01.

The data shows a steady linear decrease in P H Q 9 scores for the Social dance group, while the Waitlist group shows a less pronounced decline that plateaus between T 1 and T 3.

Navigate back to Table 3..

### Figure 2. Long description

The line graph plots Average P H Q 9 Score on the vertical Y axis, ranging from 6 to 12, against Timepoint on the horizontal X axis.

Two conditions are tracked:

* Social Dance (represented by a red line with circular markers).

* Waitlist (represented by a teal line with circular markers).

Both groups start at Eligibility with high scores around 11. At T 0 (Pre-Intervention), both groups drop to approximately 9.5.

From T 0 to T 3, the trends diverge:

* The Waitlist group shows a gradual, shallow decline, ending at T 3 (Follow-Up) with a score of approximately 8.5.

* The Social Dance group shows a steeper linear decrease, dropping to approximately 8 at T 1 (Midway), 7.8 at T 2 (Post-Intervention), and reaching its lowest point of approximately 6.5 at T 3 (Follow-Up).

Vertical error bars are present at each data point, indicating the range of variance for each measurement.

Navigate back to Figure 2..

### Figure 3. Long description

The horizontal X axis is labeled Timepoint and includes four markers: T 0 (Pre-Intervention), T 1 (Midway), T 2 (Post-Intervention), and T 3 (Follow-Up). The vertical Y axis is labeled Average Social Anxiety Score and ranges from 40 to 50.

Two lines with error bars represent the conditions:

* The Social Dance group (red line) starts at approximately 47 at T 0, shows a linear decrease to roughly 43.5 at T 2, and continues to decrease to approximately 41.5 at T 3.

* The Waitlist group (teal line) starts higher at approximately 52 at T 0 and remains relatively stable with a very slight downward trend, ending at approximately 51 at T 3.

Data points are missing for both groups at the T 1 Midway marker. The Social Dance group consistently shows lower anxiety scores and a steeper rate of improvement compared to the Waitlist group.

Navigate back to Figure 3..

### Figure 4. Long description

A two-panel figure showing happiness trends.

Left panel: Daily Happiness Scores by Condition. The x-axis represents Day from 0 to 86 and the y-axis represents Mean Happiness Score from 5 to 10. Two lines are plotted. The Social Dance line in red starts near 6 and shows a gradual upward trend with daily fluctuations, ending near 7. The Waitlist line in teal starts near 6 and remains relatively flat with higher volatility, including a sharp spike near day 45, ending near 6. Shaded areas around both lines represent standard error.

Right panel: Weekly Happiness Scores by Condition. The x-axis represents Week from Week 1 to Week 12 and the y-axis represents Mean Happiness Score from 6.0 to 6.5. The Social Dance line in red starts at approximately 6.1 in Week 1 and shows a steady increase to a peak of approximately 6.8 in Week 10 before a slight decline. The Waitlist line in teal starts at 6.0 in Week 1, drops to approximately 5.7 in Week 2, and fluctuates between 5.7 and 6.2 for the remainder of the period. Vertical error bars are present for each weekly data point.

Navigate back to Figure 4..
