# Supplementary material for: The effects of a salsa dance intervention in young people with mild to moderately severe depressive symptoms
Source: Psychol Med. 2026 Jul 15;56:e210. doi: 10.1017/S0033291726104991 (PMC13370186; doi:10.1017/S0033291726104991)
Supplement: Delattre et al. supplementary material [file S0033291726104991sup001.pdf]

Supplementary Materials: The effects of a salsa dance intervention in young people with mild to moderately severe depressive symptoms

Delattre et al., 2026

Appendix A: Patient and Participant Involvement

1. Method

The first author attended the virtual meeting of an Oxford-based Young Persons Advisory PPI group (young people aged 16-20) to explain the premise of the study and answer any initial questions the young people had. Then, following the meeting, a brief (see below) was circulated to the group members. The brief included a study description, videos of four different types of cooperative movement activities (*capoeira,* an Afro-Brazilian movement art; salsa dancing; group Scottish Cèilidh dancing; and partner and group movement "connection" games and exercises, such as mirroring activities), as well as questions for the PPI consultants to answer. Since the group happened to be comprised of only women, three men (ages 19-26) were additionally asked to read the brief, watch the videos, and respond to the questions, such that PPI study feedback was not only received from women.

2. Circulated Brief

Hello! My name is Brennan, I'm a doctoral student in Oxford's Psychiatry department, and I want to study how creative, cooperative movement can help combat loneliness and depression, especially for young people.

We think that activities that have both a social aspect and a movement-based aspect—like partner dance, where you are moving but also interacting with another person—might work even better for those experiencing depression or loneliness than solo exercise (such as going for a run by yourself) or non-movement socializing (such as group therapy).

To that end, I'd really appreciate if you can please watch the following videos, where each has a different type of "cooperative movement" that we're thinking about offering as our social movement activity in this study. These videos are of people who have already learned how to do each activity, but the classes we would offer would be welcoming to, and progressive for, people at the very beginner level.

While you watch (and when you discuss), what I'd really like to know from you is:

1. 1) If you were to imagine you and your friends, which of these do you think would appeal the most to them (and to other people your age), and why?
2. 2) What concerns might people your age have if they were invited along to a class like this?
3. 3) How would you feel if some people in the class already had a little or a lot of experience doing the activity? Would you feel more comfortable if some of the other participants in the class already had experience doing the activity, or if everyone started from the same level, having never tried the activity before?
4. 4) Any other feedback that you'd like to provide would be much appreciated!

****

Videos:
Video 1: https://www.youtube.com/watch?v=e_EcZZS7Psk&ab_channel=DanceDojo

(please start from 0:59 and watch until you get a good sense of the activity)

2. Playing Capoeira

Capoeira is an Afro-Brazilian movement art that includes elements of martial arts, dance, improvisation, and moving with a partner in physical call and response sequences, often to music. Capoeira can be more martial (like Videos 2A, 2B, and 2C) or more dance-like (like Video 2D) and I'd be very interested to know your thoughts on the more martial arts-style capoeira versus the more dance-like capoeira with respect to the questions above as well!

1. Salsa Dancing

(please watch these videos until you get a good sense of the activity)
Video 2A, 2B, and 2C: "Martial Arts" Capoeira
A: https://www.youtube.com/watch?v=dqKK7niWkFc&ab_channel=CapoeiraKarkara B: https://www.youtube.com/watch?v=uwYoexM7svQ&ab_channel=CapoeiraKarkara C: (these players are a bit more advanced!**) https://www.youtube.com/watch?v=ftbBep-**

1eSg&ab_channel=CapoeirabrasilLA

Video 2D: "Dance" Capoeira

D: https://www.youtube.com/watch?v=jGEwPtQWGkA&ab_channel=SacredMovementInst

3. Scottish Cèilidh Dancing

Participants dance together in groups and frequently switch partners / interact with multiple people.

Video 3: https://www.youtube.com/watch?v=Wm_5l_bs- 6Y&ab_channel=BBCScotland-Comedy (please start from 0:08 and watch until you get a good sense of the activity)

4. Connection Games

Connection Class, which is already offered via the University of Oxford Salsa Society, involves playing games with partners or small groups of people, including mirroring exercises, exploring embodied creativity, and gently leading and following each other in the space. These are inclusive classes that encourage experimentation and empathy, and focus on gentle and safe connection while dancing with another person or people.

Video 4: (please see video attached to this email**)**

****

If you have any problems with any of the videos or any additional questions for me before you discuss these videos and this study, please do not hesitate to contact me at brennan.delattre@psych.ox.ac.uk, and I'll do my best to assist.

Thank you very much in advance for your help with this!

Best regards,

Brennan Delattre

3. Anonymized Responses from Young People

Young Person 1 (female, age not provided, from Young Persons Advisory Group):

1) If you were to imagine you and your friends, which of these do you think would appeal the most to them (and to other people your age), and why?

After watching all the videos **either the salsa or Scottish ceilidh dancing would appeal to me the most**. I thought they both seemed really enjoyable and I enjoyed the music with both as well. I also like with the Scottish dancing it was more of a group dance and you were not with just one partner, I also liked how it appeared quite free and fun and none of the movements seemed too confusing or hard. I guess each young person is very different some people may hate the idea of a group dance and may prefer more connection games. Perhaps you could offer three different classes to young people and allow them to pick out of the three which one they want to attend to. **I know me and my friends would have the most fun doing either the salsa or Scottish dancing.** They both have fun music, and are fun to perform. I did not like the playing capoeira as much I thought it was slightly strange and the movements were a bit confusing and rigid and I would feel more uncomfortable just doing it with one person. I also know with the connection games I would maybe get anxious someone **I have never met mirroring my actions as I would be worried about what to do and what actions they would copy. I was also drawn more to the salsa an Scottish dancing as I enjoy that type of music as well.**

2) What concerns might people your age have if they were invited along to a class like this

People [may] be concerned they cannot dance, if it was a group or partnered dance what if they mess up or let the group down, people may be worried they won't remember the moves or steps, may be worried people will judge them, they may be worried they won't be fit and healthy enough for the class, they may be worried they will be the worst, also may be worried everyone will watch them performing, also some people may just not enjoy dancing.

3) How would you feel if some people in the class already had a little or a lot of experience doing the activity? Would you feel more comfortable if some of the other participants in the class already had experience doing the activity, or if everyone started from the same level, having never tried the activity before?

I personally would prefer if everyone started from the same level. I know it would be me more anxious and worried if someone was really good and I got paired with them as I would be worried how I looked compared to them. I think you could always do a beginners, middle and advanced class so new people could go in the beginners and people who have been there for a long time could go in the advanced.

4) Any other feedback that you'd like to provide would be much appreciated!

The order I would want to do them in would be **the Scottish dancing, the salsa, the connection then the capoeira**. **I love the idea of it and it seems really fun and very different from other treatments for depression.** I had a practical worry as in people may not come if it is too far from home, or if it is only offered one time each week which they cannot make. I also feel people experiencing depression often feel very low and do not have the motivation to get up and do activities so people may struggle to come or have the energy and motivation to dance. I think it would be better run in smaller groups as large groups may be overwhelming. **I think it could be really good though as people may enjoy just spending an hour a week dancing, connecting with people, making new friends and may also be a good distraction technique.**

Young Person 2 (female, age not provided, from Young Persons Advisory Group):

1) *If you were to imagine you and your friends, which of these do you think would appeal the most to them (and to other people your age), and why?*

Quite a few of my friends have anxiety, so any activity where they feel they're being perceived more than normal (like someone watching them try to figure out the capoeira) may make them uncomfortable. They'd prefer activities where they wouldn't feel awkward whilst moving, ones where the movement is already normal and fluid to them.

However, it definitely depends on the person because I'm the opposite: if I'm going to a class for something I'd want to actually learn something (like learn a dance, instead of just going running, something I can easily do in my own time), so that I don't feel like the time is being wasted.

The capoeira looks very difficult, though I think you really need to form a connection with your partner in order for the dance to work, so it could be great for the social aspect of these classes.

Ceilidh dancing seems very energetic and much easier to learn, so perhaps this is a great option for an introductory lesson? I'm not sure whether the type of dance will stay the same or change every few lessons.

2) What concerns might people your age have if they were invited along to a class like this?

- How is this actually going to help me? Maybe when the class is recommended to the individual they should be informed of all the benefits of attending (mood boosting, socialising, etc.) so they know that it has a real purpose
- Will I actually like the people there? Maybe organising a social event afterwards (like going to a café!) would encourage interpersonal connection.
- Am I going to look stupid? I think because self-perception is generally worse in depressed people, they may feel embarrassed at the thought of dancing, especially for non-dancers. If others there already have experience in the dance, I think this feeling will be heightened.
- Are there other people my age? Generally I **think keeping age groups apart is a good idea for younger people (11-15) but the groups should start mixing for older people (16-18 and 19-23+).**

3) How would you feel if some people in the class already had a little or a lot of experience doing the activity? Would you feel more comfortable if some of the other participants in the class already had experience doing the activity, or if everyone started from the same level, having never tried the activity before?

If others were already experienced in the activity, I'd feel like I was "behind" even before the classes even started.

By having everyone start at the same level, it lowers the mental barriers to entry and will probably make the experience much more enjoyable for everyone involved- everyone can progress together!

Maybe there could be different classes for different skill levels? So that way we aren't excluding anybody for being more/less experienced.

4) Any other feedback that you'd like to provide would be much appreciated!

Perhaps the classes could diverge from only dance lessons- whilst this would work for some people, others aren't great at physical co-ordination so may end up with more frustration than enjoyment. For these individuals, maybe an endurance sport or a group yoga sessions would be more beneficial.

Connection is a very vague name for the class. Maybe instead of changing the name, you could give a 1-sentence explanation of what it is and what the function of it is.

Just an additional thought: will the individuals pick their partners or will they be assigned partners? There's definitely pros and cons for either option.

Young Person 3 (male, 19, transcript of voice recording):

"I think in terms of question one ["If you were to imagine you and your friends, which of these do you think would appeal the most to them (and to other people your age), and why**?"], salsa dancing is something that I feel like could appeal to the most people, regardless of whether they're my peers or not, just because it's a universal thing - salsa dancing is something a lot of people have fun with. It's really easy to connect with people, and it's just fun**. And to that end I feel like it wouldn't really matter if your partner or peers in this case have more experience than you, just cuz, like, I think it's fun, to have someone who - I remember my first time I was salsa dancing with my friend's mom, and she was like **obviously way better than I was (she's Latin American and she's in her thirties and I was like kind of outclassed) but that's kind of what made it fun, and the interaction you have is fun, improvising, getting to know your partner**, I think it's really a cool experience. And I guess people my age, I feel like their only concern would be - I don't know, just getting out there, and making a new connection, maybe? I mean like it's always a little stressful (daunting), if the setting is how I'm envisioning it, and you're in a dance class with a bunch of people you've never met, it could be kind of daunting to be like "hey you, dance with me" without knowing [them yet], but yeah, I **feel like a lot of my friends would be down to go salsa dancing**, because yeah, it's fun."

Young Person 4 (male, 20):

1) If you were to imagine you and your friends, which of these do you think would appeal the most to them (and to other people your age), and why?

Playing Capoeira, because it incorporates martial arts, which is extremely popular in today's UFC fandom culture. Capoeira is also a very sense-based form of martial art/dance that connects you to all of your bodies muscles, and I believe it to be the most meditative for me personally out of these. Meditation is gaining more and more importance in today's world with more screens and social media for young adults to deal with it creates more anxiety for everyone.

2) What concerns might people your age have if they were invited along to a class like this?

As far as salsa dancing goes, I think most people would be nervous at first but eventually would have a fantastic experience. If you have never danced in that setting before it is the purest form of being outside your comfort zone. That can be a great thing for people who don't get out often or want to meet new acquaintances.

3) How would you feel if some people in the class already had a little or a lot of experience doing the activity? Would you feel more comfortable if some of the other participants in the class already had experience doing the activity, or if everyone started from the same level, having never tried the activity before?

I think it would be encouraging to everyone in the class if they were at the same level. It never feels good when the Instructor or teacher has a favorite student purely because of their skill level. Another benefit of having the same level of experience as your peers is that you have nothing to lose and everything to gain. Everybody is in the same boat as you so there's no reason to be scared of failure and the only way to go is up!

Young Person 5 (male, 26, history of depression and social isolation):

1) If you were to imagine you and your friends, which of these do you think would appeal the most to them (and to other people your age), and why?

The martial side of capoeira appeals to me the most out of all of them, probably because there's the potential to progress to higher level/difficulty sequences/moves, and because it offers some physical benefit outside of just cardio like the others.

2) What concerns might people your age have if they were invited along to a class like this?

Salsa: Being more based in [a rural UK town rather than Oxford], I can see people being concerned about displaying lack of skill, rhythm, coordination, sequence memory, etc, or **some of the really conservative minded not wanting to appear un-masculine**. More generally I can see people my age maybe having reservations based on the proximity and physicality with a partner, **as some people just aren't that comfortable being touched, especially by strangers, and could otherwise cause a bit of social anxiety**. Lastly, seeing as salsa is "traditionally" a mixed activity I can see people having reservations about the intimacy of the dance (not in a religious sense), though again, I don't see this being as much of an issue in places like Oxford over [the rural UK town in which I live].

Capoeira: Balance, flexibility, and core strength for sure, especially for the hand/head stands. **Memorising the sequences also seems more daunting than salsa, but it's rewarding too.** Since it's non-contact and not traditionally mixed it escapes any physical touch or intimacy reservations. Lastly, I can see leg flexibility and hand/head stands being daunting for overweight people, especially since long term depression can cause weight gain.

Ceilidh: could see some people, particularly gen Z from cities, **viewing it as old fashioned or for old people, which could affect the numbers who turn up, which is more problematic for this kind of dance I think than others.** Otherwise, the fact you dance in groups and only split into partners momentarily alleviates some of the social anxiety issues I mention in Salsa, and the simplicity also helps with sequence memory.

Connection games: **lower impact level makes its entry level good for all fitness levels**. No touching in the video **but there is a level of intimacy that some may find awkward, although I'm guessing when comes to addressing loneliness that's needed right? the softer nature could be more appealing to people after a long day than something spirited like capoeira**, but that softer side also means it has less of a repeatability to it, since the activity doesn't advance so much as just change.

3) How would you feel if some people in the class already had a little or a lot of experience doing the activity? Would you feel more comfortable if some of the other participants in the class already had experience doing the activity, or if everyone started from the same level, having never tried the activity before?

I'd personally find having more experienced members helpful, since you can get some guidance from them. that **said, having some dedicated moments for newcomers within a session would help people from struggling or feeling like they're in the deep end,** especially for the one-on-one activities (barring connection games which have low barrier to entry) where there as more focus on both people playing a part to enable their partner.

Appendix B: Reasons Provided for Discontinuing or Partially Completing the Study

Of the participants that did not finish the study, eleven participants became too busy or reported too many academic commitments to continue; eight participants stopped attending classes and/or stopped responding to the research team; one had an unrelated ankle injury; one could not attend classes due to illness; one unenrolled due to not being randomly assigned to the same group as a friend in the study; one unenrolled due to a history of undiagnosed disordered eating behaviors that were not mentioned to the research team during screening and developing the worry that a dance-based activity could contribute to a potential relapse; one was unenrolled by the research team for participating in two similar studies simultaneously; one unenrolled because they did not feel comfortable going back by themself after their friend dropped out; and two did not specify a reason.

Additionally, completing the experimental condition required attending at least six out of eight classes. Three experimental group participants completed all the questionnaire and task measures but only attended four or five classes due to illness (n = 2) or moving out of the city (n = 1). These participants were counted as study completers.

Appendix C: Linear Mixed Models for Loneliness (UCLA-L) and Anxiety (GAD-7)

Loneliness (UCLA-L Scores)

Two LMMs were conducted to examine the effect of condition (social dance vs. waitlist) and time point (T0, T1, T2, T3) on loneliness, using both the UCLA-3 item and direct-item measures. Models included random intercepts for participant IDs to account for individual variability. With respect to the 3-item scale, results revealed a significant main effect of time point, with significant decreases in UCLA-3 item scores at T1 (B = -9.92, SE = 0.20, t = -4.53, p < .001), T2 (B = -0.92, SE = 0.20, t = -4.53, p < .001); and T3 (B = -1.22, SE = 0.20, t = -6.04, p < .001) compared to T0, indicating an overall decrease in loneliness over time. However, the main effect of condition was not significant (B = 0.18, SE = 0.33, t = 0.55, p = .58), suggesting that the waitlist condition did not significantly differ from the social dance condition. The interaction between condition and time point, while not significant, approached significance for T3 only (B = 0.47, SE = 0.30, t = 1.60, p = .110, indicating a possible differential effect of the intervention at later time points. With respect to the direct loneliness item, there was again a significant main effect of time point, with a significant decrease in scores at T3 (B = -0.51, SE = 0.14, t = -3.56, p = .0004). There was again no main effect of condition (B = 0.24, SE = 0.20, t = 1.18, p = .24) nor interaction between condition and time point. While there was an overall significant reduction in loneliness over time in study participants on both loneliness measures, there was no significant effect of condition; additionally, while the interaction between condition and time point approached significance for the 3-item loneliness measure at T3, indicating a potential difference between conditions later in time, further research would be needed to clarify this trend.

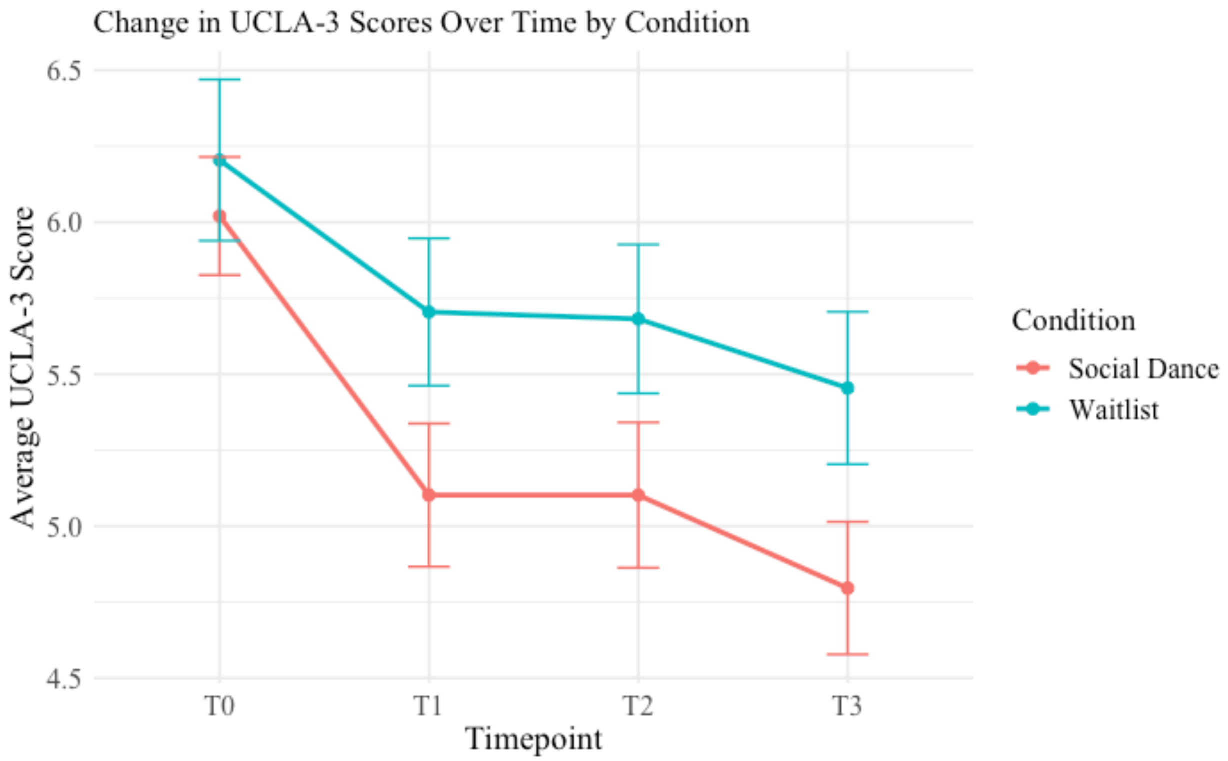

Figure S1. Change in UCLA-3-item scores over time by condition. Higher scores indicate more loneliness. *N = 93*.

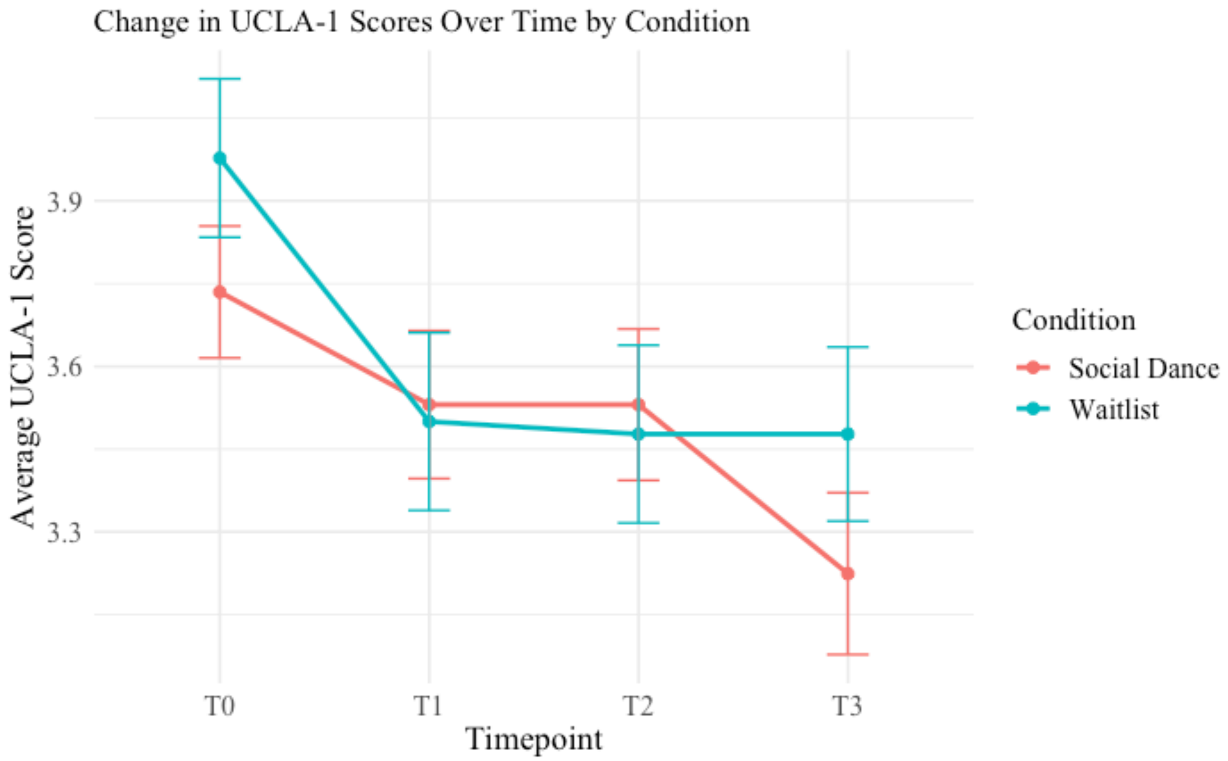

Figure S2. Change in UCLA direct item scores over time by condition. Higher scores indicate more loneliness. *N = 93*.

Anxiety (GAD-7 Score)

An LLM was conducted to examine the effects of condition and time point on GAD-7 score, with participant ID included as a random intercept. The model showed a significant main effect of time point, indicating that GAD-7 scores decreased over time. Compared to T0, scores were significantly lower at T1 (B = -1.39, SE = 0.58, $t(273)$ = -2.38; p = .018); T2 (B = -1.69, SE = 0.58, $t(273)$ = -2.91, p = .004; and T3 (B = -2.65, SE = 0.58, $t(273)$ = -4.55, p < .001). However, there was no main effect of condition (social dance vs. waitlist), B = 0.07, p = .94, suggesting no overall difference in GAD-7 scores between groups. There was also no significant condition by time point interaction (**T1:** B = 1.12, SE = 0.85, $t(273)$=1.32, p = .19; **T2**: B = 0.88, SE = 0.85, t (273)=1.03, p = .30; **T3:** B = 1.31, SE = 0.85, $t(273)$ = 1.55, p = .12), suggesting that the rate of GAD-7 score reduction over time did not significantly differ between the social dance and waitlist conditions. Additionally, the random effects indicated substantial variability in baseline anxiety across participants, with a random intercept variance of 10.8 (SD = 3.29), indicating considerable individual differences in GAD-7 scores.

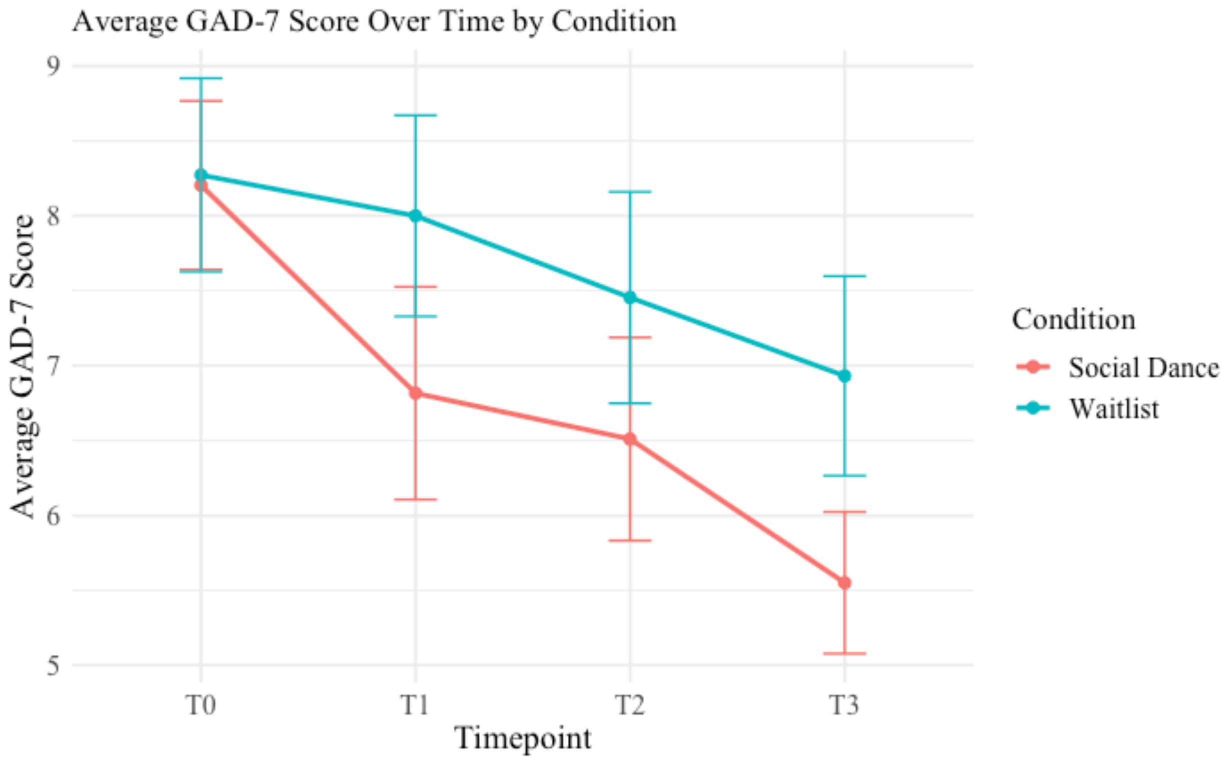

Figure S3. Change in GAD-7 scores over time by condition. N = *93.*

Appendix D: Empathy and Post Class Enjoyment / Positive Class Experience as Moderators

Basic Empathy Score as a Moderator of PHQ-9 Changes (all participants)

A linear mixed-effects model was conducted to examine whether participants' average empathy score moderated change in PHQ-9 scores across four timepoints. The model included random intercepts for participants to account for repeated measures. There was a significant main effect of average empathy score, $B = 0.19$, $SE = 0.10$, $t(92.00) = 2.00$, $p = .049$, indicating that individuals with higher empathy scores had slightly higher overall PHQ-9 scores. However, the interaction between average empathy score and timepoint was not significant at any order (linear, quadratic, cubic, quartic; all $p > .17$), suggesting that changes in depression scores over time did not differ as a function of empathy.

Another linear mixed-effects model was conducted to examine whether participants' average empathy moderated the effect of condition (social dance vs. waitlist) on PHQ-9 scores across the four timepoints. Random intercepts were again included for participants. There were no significant main effects of condition, $B = -13.71$, $SE = 12.67$, $t(90.00) = -1.08$, $p = .282$, or average empathy score, $B = 0.14$, $SE = 0.15$, $t(90.00) = 0.92$, $p = .360$. Furthermore, no two-way or three-way interactions involving timepoint, condition, and average empathy score were significant (all *ps* > .23), indicating that empathy did not moderate the impact of condition on depression symptoms over time.

Post Class Enjoyment Measures – Positive Class Experience

Pearson correlation analyses were conducted to assess the relationship between the three post-class Likert-scale variables—*How enjoyable did you find today's class?* ("Enjoyment"), *How connected to others do you feel right now?* ("Connected"), and *How happy do you feel right now?* ("Happy")—for all participants across time points. Enjoyment was significantly correlated with Connected, $r(275) = 0.64$, $p < .001$; and with Happy, $r(276) = .59$, $p < .001$. Connected and Happy were also significantly correlated, $r(275) = .72$, $p < .001$. These strong, positive associations suggested that these variables may reflect a common underlying construct related to participants' subjective class experience.

To further evaluate the internal consistency between these three variables, Cronbach's alpha was calculated. The three items demonstrated excellent internal consistency, $\alpha = .85$, 95% CI [.81, .88], with an average inter-item correlation of $r = .65$. Item-total correlations ranged from $r = .72$ to .83, and the scale mean was 7.57 ($SD = 1.34$). These results indicate that the three items are highly interrelated and can be considered indicators of a common latent factor. Given the strong correlations and high internal consistency, the three variables were combined into a single composite variable by taking the mean score of the three variables per participant; this new variable was termed Positive Class Experience.

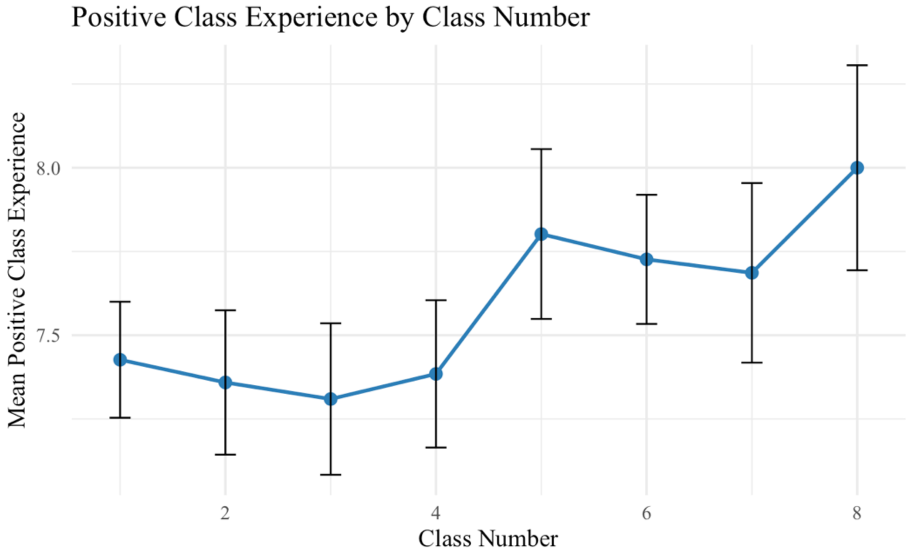

Figure S4. *Positive Class Experience as a Moderator of PHQ-9 changes (social dance participants only)*

A linear mixed-effects model was conducted to examine whether positive class experience moderated changes in PHQ-9 scores across four timepoints (T0–T3) among participants in the social dance condition. Participant ID was included as a random intercept to account for repeated measures. There was no significant linear effect of time, $B = -0.32$, $SE = 3.16$, $t(188) = -0.10$, $p = .920$, but both quadratic and cubic time effects were significant, $B = -12.92$, $SE = 3.16$, $t(188) = -4.10$, $p < .001$, and $B = -7.82$, $SE = 3.16$, $t(188) = -2.48$, $p = .014$, respectively. Importantly, positive class experience significantly moderated the quadratic effect of time, $B = 1.78$, $SE = 0.42$, $t(188) = 4.28$, $p < .001$, and the cubic effect, $B = 0.97$, $SE = 0.42$, $t(188) = 2.34$, $p = .020$. These

interactions suggest that participants who reported more positive experiences in class exhibited different patterns of change in depressive symptoms across time.

To examine these patterns, social dance participants were median-split into those that had more positive (n = 25, M = 8.22, SD = 0.49, range = 7.52-9.29) and less positive (n = 24, M = 6.80, SD = 0.78, range = 4.11-7.5) experiences in salsa dance classes. Then, PHQ-9 score over timepoints was plotted by more and less positive class experience to examine the amount of change and curvature differences between the two groups.

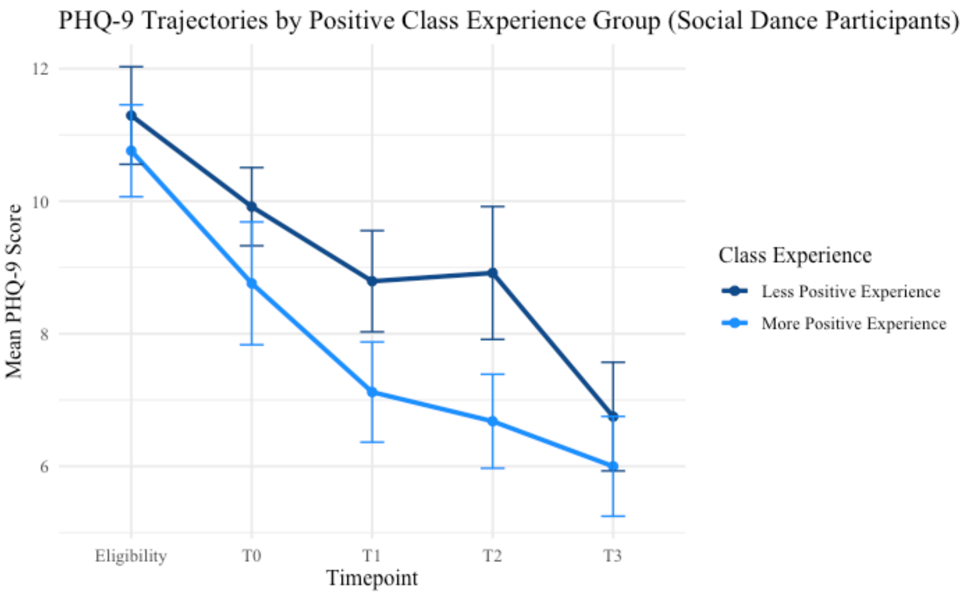

Figure S5. PHQ-9 Trajectories by Positive Class Experience Group (Social Dance Participants)

Appendix E: Sensitivity Analysis with Missing Daily Mood Data

To evaluate whether missing data influenced results, sensitivity analyses excluding participants with more than 60%, 70%, or 80% missing daily or weekly mood ratings were conducted. Model estimates were highly consistent across thresholds for both daily and weekly data. For daily mood, the fixed effect of condition (social dance vs. waitlist) showed t-values ranging from -1.48 to -1.77, the effect of day remained significant (ts = 8.60–8.71), and the condition × day interaction varied minimally (ts = -3.16 to -3.20). Similarly, for weekly mood, the condition effect ranged from t = -0.46 to -0.03, and other fixed effects such as day and interactions showed stable t-values ranging approximately from 1.63 to 6.17. Standard errors across all models differed by less than 5%. These findings indicate that participants with high levels of missingness did not meaningfully influence results. Because linear mixed-effects models are robust to missingness under the assumption that data are missing at random (MAR), all participants were retained in these analyses to maximize power and generalizability.

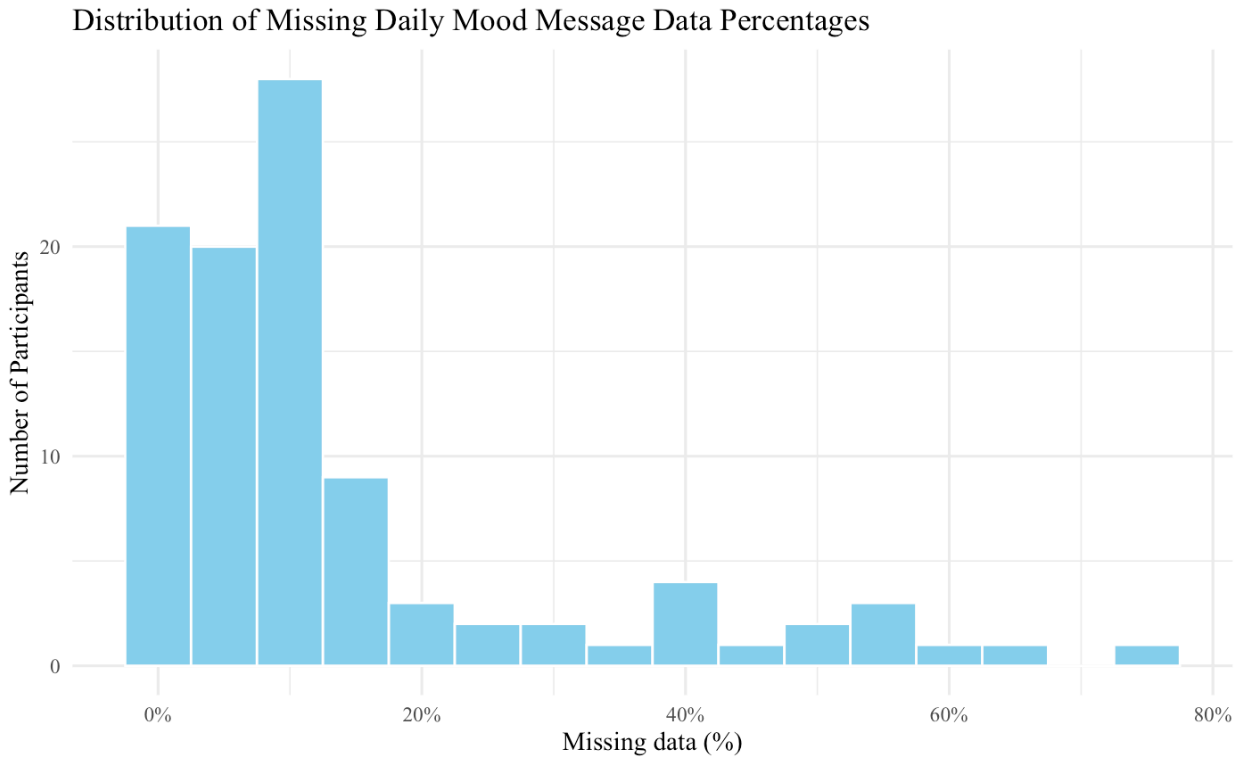

Figure S6. Distribution of Missing Daily Mood Message Data Percentages.

Appendix F: Social and Emotional Processing Tasks

Social and emotional processing (SEP) and social interaction task outcome measures were included alongside participant self-report and assessed before, during, and following the social movement sessions compared to the waitlist control to help elucidate the potential mechanisms underlying changes in clinical and subjective experience. SEP tasks, such as emotional facial recognition and memory for emotional words, have been demonstrated to correspond with early cognitive changes that can be predictive of mood changes and treatment efficacy downstream (Harmer et al., 2017).

We anticipated that improvements in social and emotional functioning and reductions in potentially disrupted social learning behavior, as demonstrated via one or several of these tasks, could assist in elucidating the possible mechanisms responsible for mood improvement from social movement, if mood improvement was seen.

Specifically, four tasks from the Oxford Emotional Task Battery (ETB) were used to examine changes to emotional facial recognition, emotional word categorization, and emotional memory from before to after the social dance intervention versus the waitlist control; these tasks have been demonstrated to have good test-retest reliability (Thomas et al., 2016). These tasks were administered online via Gorilla Experiment Builder (www.gorilla.sc; Anwyl-Irvine et al., 2018), and the updated words version was used (Raslescu et al., 2023). As the ETB has been found to be sensitive to the early effects of antidepressant administration on emotional processing (specifically, negative affect bias) preceding clinical changes to mood (Harmer et al., 2009), these tasks were selected to investigate potential emotional processing changes that could precede mood changes in response to this intervention. These tasks were administered at baseline and T2. While the first author was not blinded, data quality exclusion decisions were made by academic supervisors CH and SM prior to unblinding task data for experimental vs. control group analyses.

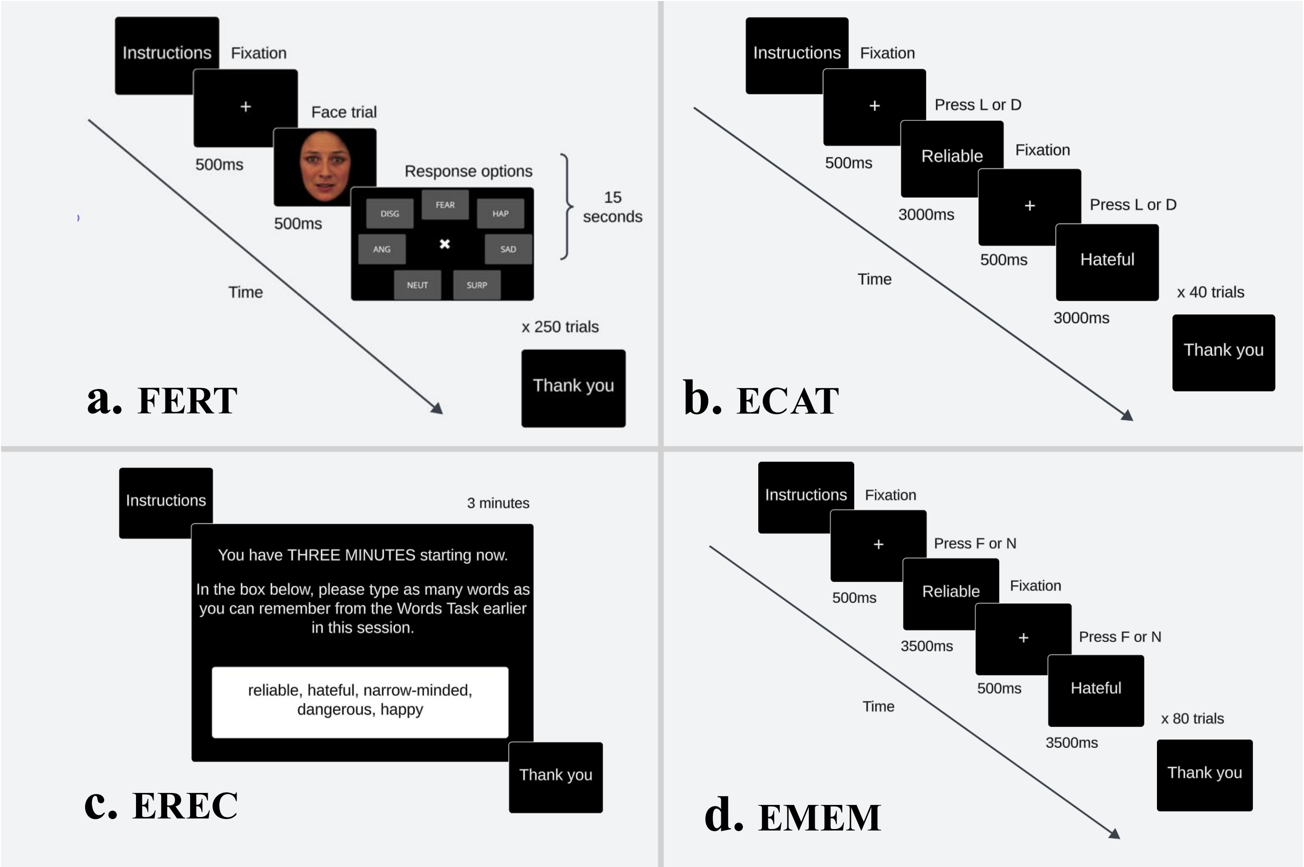

Figure S7. (a) FERT; (b) ECAT; (c) EREC; (d) EMEM. *Images from Raslescu, 2025.*

Emotional Facial Recognition (FERT Task)

The primary emotional processing task in the ETB is the Facial Expression Recognition Task (FERT), in which participants must identify the expression of faces presented very quickly on screen (Raslescu, 2025). Task facial expressions are happiness, sadness, fear, anger, disgust, surprise, or neutral, and the degree of emotion shown by each face can vary from 10% to 100% intensity, in steps of 10%. Participants must click the correct expression-labelled button to report the expression. Each face is presented for 500ms, and participants have 15 seconds to make a response. This version of the FERT had a total of 250 trials divided into four blocks, with breaks at 25%, 50% and 75% completion. Faces were presented in the same fixed, pseudo-randomised order to all participants, with emotions, intensities and actors balanced across blocks (Raslescu, 2025; Figure S7a). The one trial below 200ms (T2) was removed.

Mean percent identification accuracy for positive and negative facial expressions

Accuracy for positive and negative facial expressions was analyzed using linear mixed-effects models with condition (social dance vs. waitlist) and timepoint (T0 vs. T2) as predictors and random intercepts for participants. For positive expressions, there were no significant effects condition, $b = 1.74$, $SE = 1.49$, $t(158.32) = 1.17$, $p = .245$, timepoint, $b = –0.57$, $SE = 1.14$, $t(91) = –0.50$, $p = .616$, or a condition × timepoint interaction, $p = .713$. For negative expressions, there was a significant main effect of timepoint, $b = –3.31$, $SE = 1.07$, $t(91) = –3.09$, $p = .003$, such that accuracy for negative expressions decreased from T0 to T2 across groups. There was no significant main effect of condition, nor significant condition × timepoint interaction, $ps > .78$.

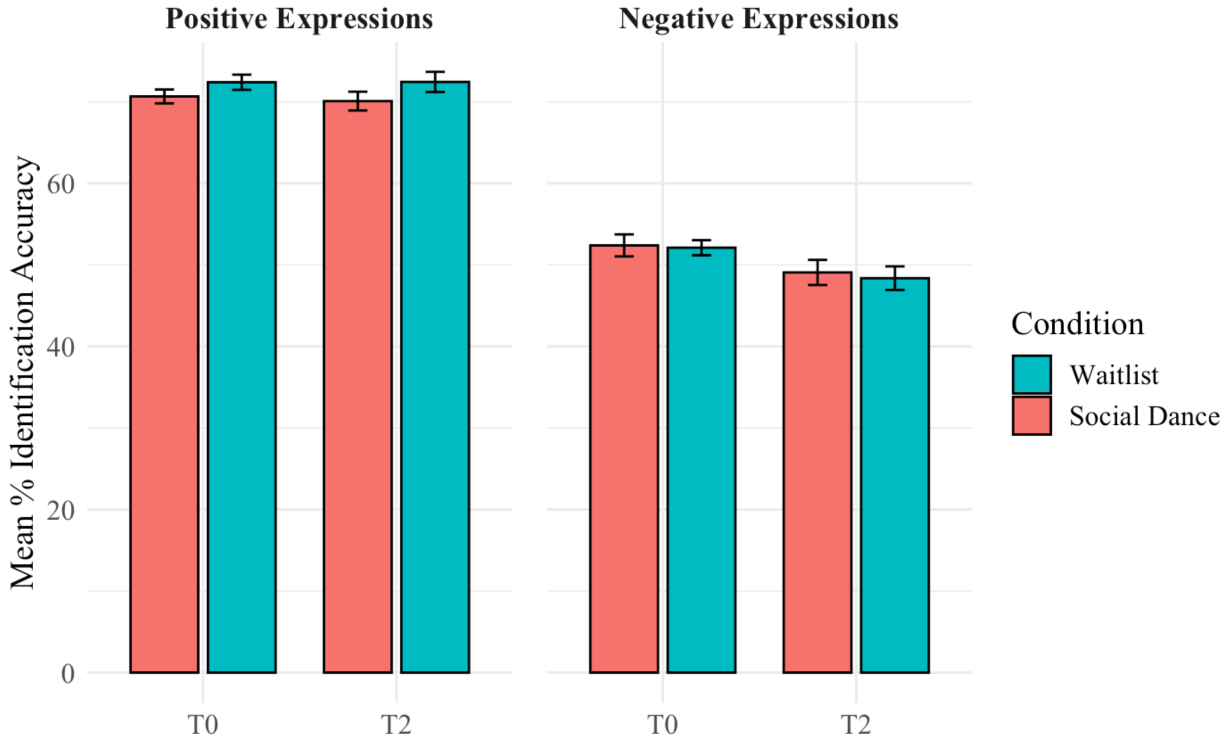

Figure S8. Participants' accuracy (%) for classifying positive facial expressions (happiness, surprise) versus their accuracy (%) for negative facial expressions (anger, disgust, fear, sadness) by experimental condition and time point.

Mean misclassifications for positive and negative facial expressions

Misclassifications for positive and negative facial expressions overall were analyzed with linear mixed-effects models with condition (social dance vs. waitlist) and timepoint (T0 vs. T2) as predictors and random intercepts for participants. There were no significant main effects of

condition, timepoint, or condition by timepoint interaction for either positive (*ps* >.308) or negative (*ps* >.145) facial expressions.

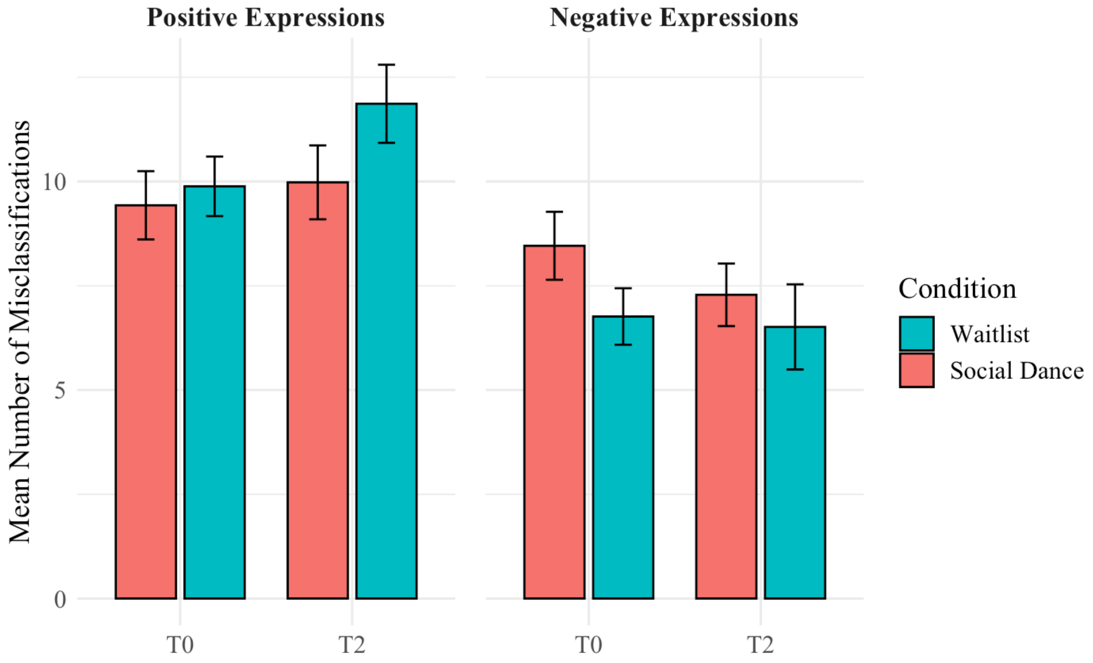

Figure S9. Participants' mean number of misclassifications (false alarms) for positive facial expressions (happiness, surprise) versus their accuracy (%) for negative facial expressions (anger, disgust, fear, sadness) by experimental condition and time point.

Reaction time (ms) for positive and negative facial expressions

Reaction times for positive and negative facial expressions were also analyzed with linear mixed-effects models with condition and timepoint as predictors and random intercepts for participants. For positive expressions, there was a significant main effect of condition, $b = –221.25$, $SE = 81.34$, $t(135.59) = –2.72$, $p = .007$, indicating that participants in the social dance condition responded more quickly to positive faces than those in the waitlist condition. The main effect of timepoint was not significant, $p = .100$, and the condition × timepoint interaction was not significant, $p = .784$. For negative expressions, reaction times were significantly faster at T2 compared to T0, $b = –144.83$, $SE = 48.92$, $t(91.00) = –2.96$, $p = .004$. Additionally, participants in the social dance condition responded significantly faster overall than those in the waitlist condition, $b = –184.94$, $SE = 81.77$, $t(131.27) = –2.26$, $p = .025$. However, the condition × timepoint interaction was again not significant, $p = .455$.

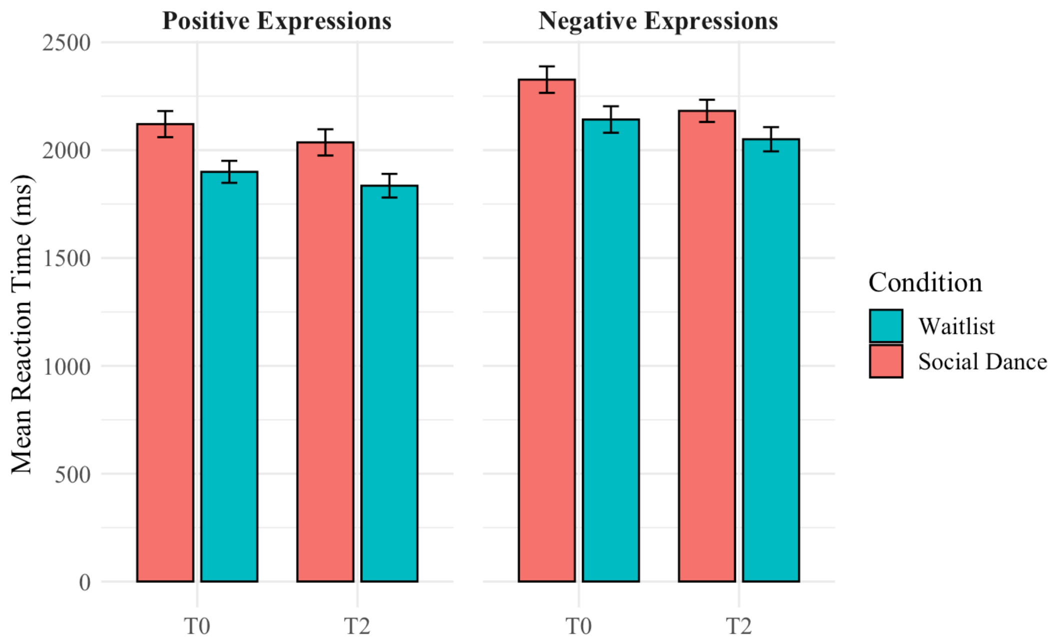

Figure S10. Participants' mean reaction times for classifying positive facial expressions (happiness, surprise) versus their accuracy (%) for negative facial expressions (anger, disgust, fear, sadness) by experimental condition and time point.

Emotional Word Categorization (ECAT Task)

In the Emotional Categorization Task (ECAT), participants had to decide whether they would like or dislike to be described as different personality characteristic words, both positive and negative, imagining a context where they have overheard a third party describing them in that way. These 40 words—20 positive and 20 negative—were presented for three seconds each. Participants entered their responses with the "D" key for disliking being associated with the descriptor and the "L" key for liking being associated with the descriptor. ECAT words were presented in a randomized order that is refreshed for each participant (Raslescu, 2025; Figure S7b). One participant's baseline ECAT data was excluded completely for having too many timeouts at the baseline visit (n=18), and another participant's baseline ECAT data was excluded for clearly being an outlier in terms of low accuracy. Any trials where the reaction time was faster than 200ms were removed (n = 3 trials at T0, and n = 1 trial at T2).

To examine whether reaction times (RTs) to classify emotional words changed over time as a function of condition and word valence, paired-sample t-tests were conducted for each Valence × Condition combination. For participants in the social dance condition, there was no significant

difference in RTs between timepoints for negative stimuli, *t(*46) = 0.17, *p* = .87, or for positive stimuli, *t*(46) = 0.09, *p* = .93. Similarly, in the waitlist condition, RTs did not differ significantly between timepoints for negative stimuli, *t*(43) = 0.09, *p* = .93, or positive stimuli, *t*(43) = 0.25, *p* = .81. These results suggest that reaction times did not change over time and were not significantly influenced by the intervention condition.

Next, to investigate whether accuracy in classifying emotional words changed over time as a function of condition and word valence, paired-sample t-tests were again conducted for each valence x condition combination. For social dance participants, there was no significant difference in accuracy over time for negative words (*t*(46) = 0.08, *p* = .94, 95% CI [-2.64, 2.85]) and the increase in accuracy for positive words was not statistically significant (*t*(46) = 1.57, *p* = .12, 95% CI [-0.39, 3.17]). For waitlist participants, there was also no significant change in accuracy for classifying negative words (*t*(43) = -0.54, *p* = .59, 95% CI [-2.12, 1.22]), nor was the increase in accuracy for classifying positive words statistically significant (*t*(43) = 1.85, *p* = .071, 95% CI [-0.12, 2.66]).

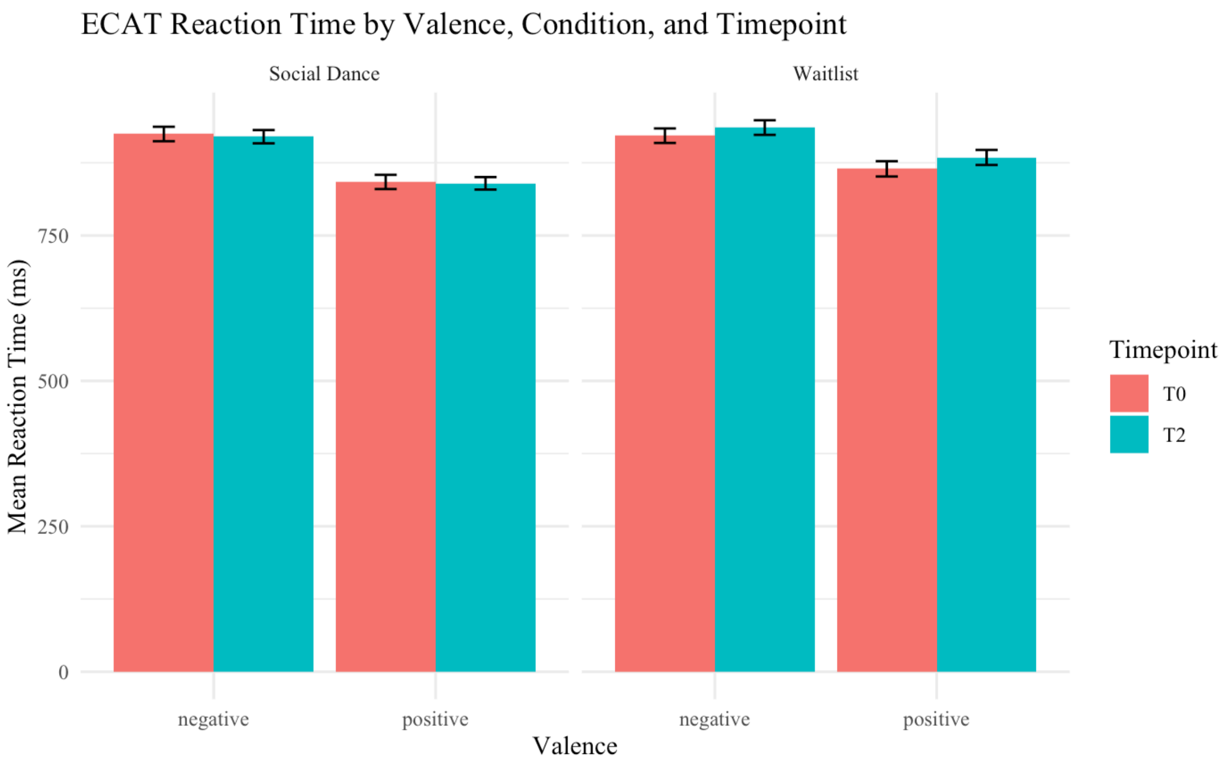

Figure S11. ECAT Reaction Time by Valence, Condition, and Timepoint.

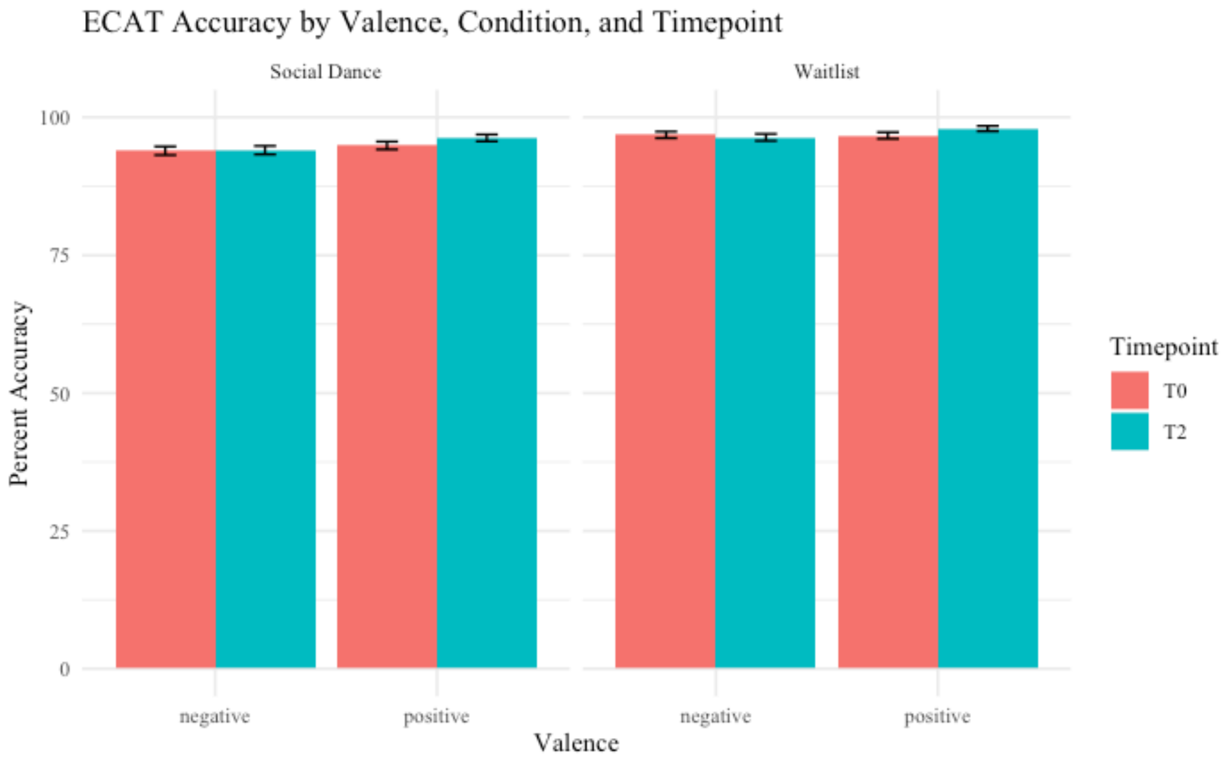

Figure S12. ECAT Accuracy by Valence, Condition, and Timepoint.

Emotional Word Recognition (EREC Task)

In the Emotional Recall Task (EREC), participants had to recall as many words as they could of the words presented during the ECAT in three minutes by typing them into a box (Raslescu, 2025; Figure 7c). For each participant, the number of positive and negative words correctly recalled, as well as the number of positive and negative "false alarms"—words recalled that were not on the original list presented—were tabulated. False alarm words were double rated by Brennan Delattre and Ella Williams to ensure agreement on word valence. Permutations of words (e.g., "free-thinking" instead of "freethinking", or "disorganized" instead of "disorderly") were accepted if they were close in spelling and had the same or a similar meaning to the original word. Twelve unique words were classified as uncategorizable for not having either a clear meaning (n=2) or a clear valence (n=10 words) and removed from analyses. Any "I don't remember any more words, sorry!" phrases or equivalent were removed. Any second instances of duplicate words (if a participant wrote the same word twice) were removed (n=0 words at baseline and n=2 words at EOS). Words recalled from the three-word pre-task practice set were not counted as false alarms but instead removed from analysis (n=64 instances across words at baseline; n=66 instances across words at end of study).

One participant's baseline EREC data was excluded completely based on having too many timeouts on the ECAT at the baseline visit (*n*=18), and another participant's EREC data was excluded because their baseline ECAT data was excluded for clearly being an outlier in terms of low accuracy; and one participant's data was removed as they wrote no words during the EREC baseline.

Correct Words ("Hits"): A linear mixed-effects model was conducted to examine the effects of condition, timepoint, and their interaction on the number of correct positive words recalled with random intercepts for participants to account for repeated measures. There was no significant main effect of condition, $b = -0.15$, $SE = 0.50$, $t(152) = -0.31$, $p = .76$, nor timepoint, $b = -0.63$, $SE = 0.38$, $t(88) = -1.64$, $p = .11$, nor a significant interaction, $b = 0.06$, $SE = 0.55$, $t(88) = 0.11$, $p = .91$. A similar linear mixed-effects model was conducted for correct negative words, and again there were no significant effects (Condition: $b = 0.32$, $SE = 0.48$, $t(146) = 0.67$, $p = .51$; timepoint, $b = 0.04$, $SE = 0.35$, $t(88) = 0.13$, $p = .90$, or their interaction, $b = -0.70$, $SE = 0.50$, $t(88) = -1.41$, $p = .16$).

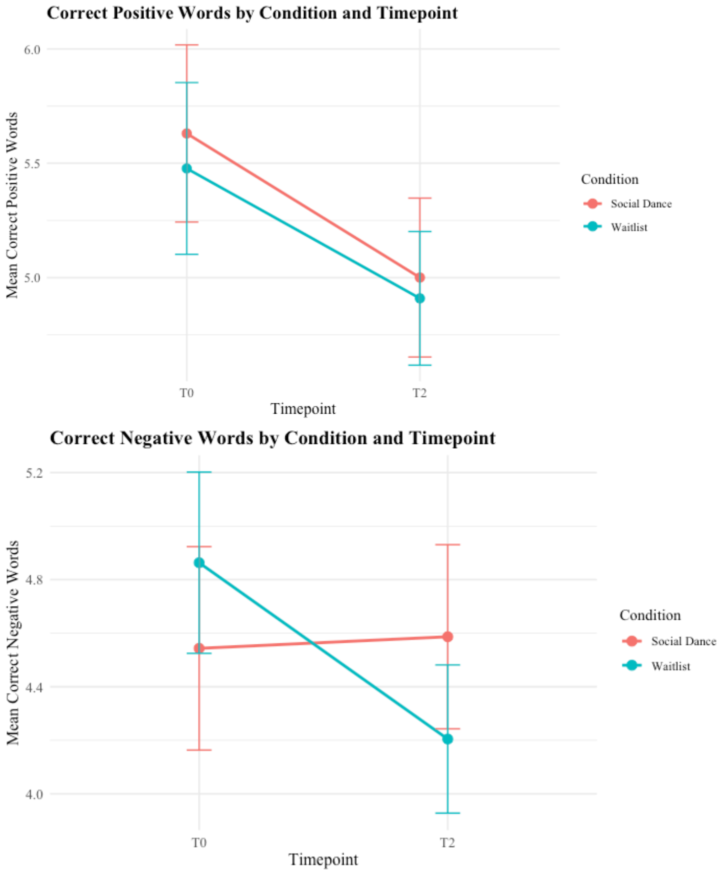

Figure S13. Correct Positive and Negative Words by Condition and Timepoint.

False alarms:

To examine whether positive and negative intrusions differed by condition and timepoint, two linear mixed-effects models were conducted—one for positive intrusions and one for negative intrusions—with condition, timepoint, and their interaction as fixed effects and participant ID as a random intercept. For positive intrusions, there was no significant effect of condition, $b = -0.58$, $SE = 0.39$, $t(162.47) = -1.46$, $p = .145$, timepoint, $b = -0.33$, $SE = 0.33$, $t(88) = -1.00$, $p = .323$, or interaction between condition and timepoint, $b = 0.26$, $SE = 0.47$, $t(88) = 0.55$, $p = .584$. While the negative intrusions model revealed a significant main effect of condition, $b = -0.44$, $SE = 0.22$, $t(126.10) = -1.99$, $p = .049$, indicating that participants in the waitlist condition reported fewer negative intrusions on average across timepoints, there was no significant main effect of timepoint, $b = 0.09$, $SE = 0.13$, $t(88) = 0.65$, $p = .518$, nor a significant interaction of condition and time, $b = 0.21$, $SE = 0.19$, $t(88) = 1.09$, $p = .280$.

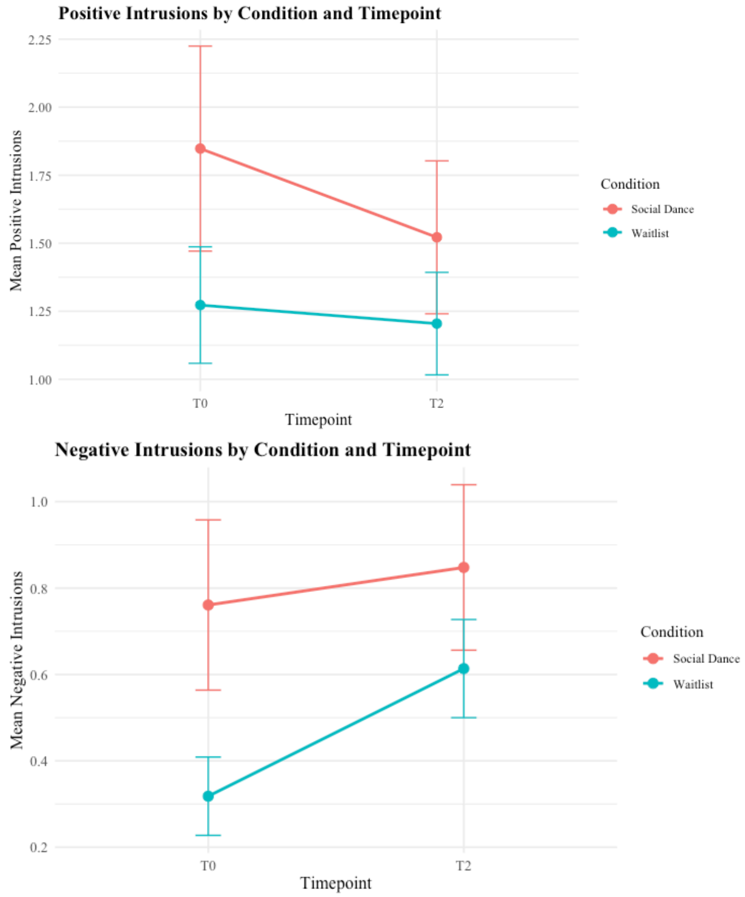

Figure S14. Positive and Negative Intrusions by Condition and Timepoint.

Memory for Emotional Words (EMEM Task)

In the Emotional Memory Task (EMEM), participants must indicate whether words presented on the screen were presented during the ECAT ("familiar") or not ("novel" distractor words). The EMEM contains 80

words (40 familiar and 40 novel), and each are presented for 3.5 seconds. Participants respond by pressing the “f” key for familiar, or the “n” key for novel. The 80 EMEM words are presented in randomized order, which is again refreshed for each participant (Raslescu, 2025; Figure S7d).

One participant’s baseline EMEM data was excluded completely based on having too many timeouts on the ECAT at the baseline visit (n=18), and another participant’s EREC data was excluded because their baseline ECAT data was excluded for clearly being an outlier in terms of low accuracy. Any trials where the reaction time was faster than 200ms were removed. Percentage identification accuracy was computed for positive familiar words, positive novel distractor words, negative familiar words, and negative novel distractor words at each timepoint and for each experimental condition (Figure S15).

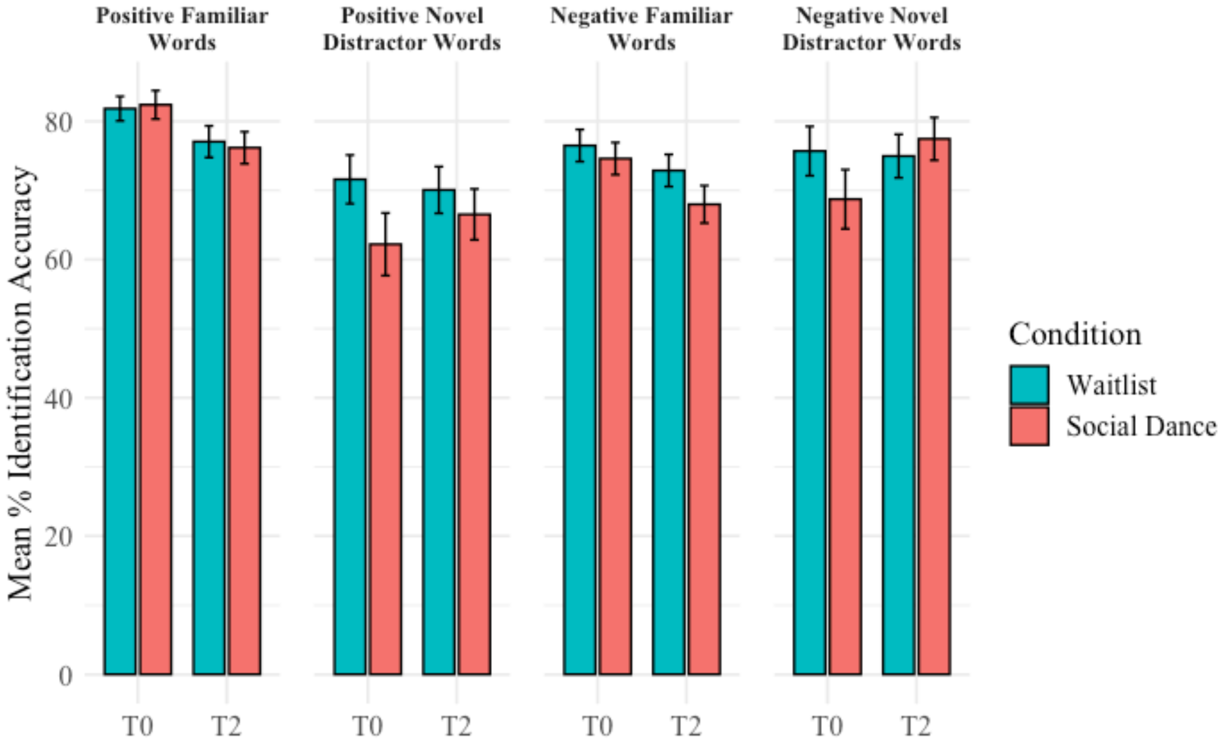

Figure S15. Mean percentage identification accuracy by word type for the EMEM.

Linear mixed-effects models predicting percent identification accuracy by condition and time point (with random intercepts for participants) were conducted.

For negative familiar words, there were no significant main effects of condition or timepoint and no significant interaction between condition and timepoint. For negative novel distractor words, there were again no significant main effects of condition or timepoint, but the condition by timepoint interaction approached significance, $b = 9.44$, $SE = 4.80$, $t(91) = 1.97$, $p = .052$, suggesting a potential trend for the effect of timepoint on accuracy that differed by condition that merits further investigation. For positive

novel distractor words, there were again no significant main effects or condition by timepoint interaction. For positive familiar words, there was a significant main effect of timepoint, $b = -4.77$, $SE = 2.13$, $t(91) = -2.24$, $p = .028$, indicating lower identification accuracy at T2 than at T0, but there was no significant main effect of condition, nor a condition by timepoint interaction.

Social Learning - Generosity Task

The Social Learning - Generosity Task (SL-Gen) is used to investigate the role of social learning and generosity sensitivity with a social exchange game-based task (Delattre et al., 2026). This task was administered at baseline and T2.

Outcome measures

Total points earned or lost in the game

This variable is calculated as the net points participants earned or lost in the game, which is their final point score minus 200 points (the number of points participants would earn in the game if they made no investments). A linear mixed-effects model showed no significant effects of Timepoint, Condition, or their interaction on points earned in the task (all $p > .34$), indicating no differences in points gained or lost over time or between social dance and waitlist groups.

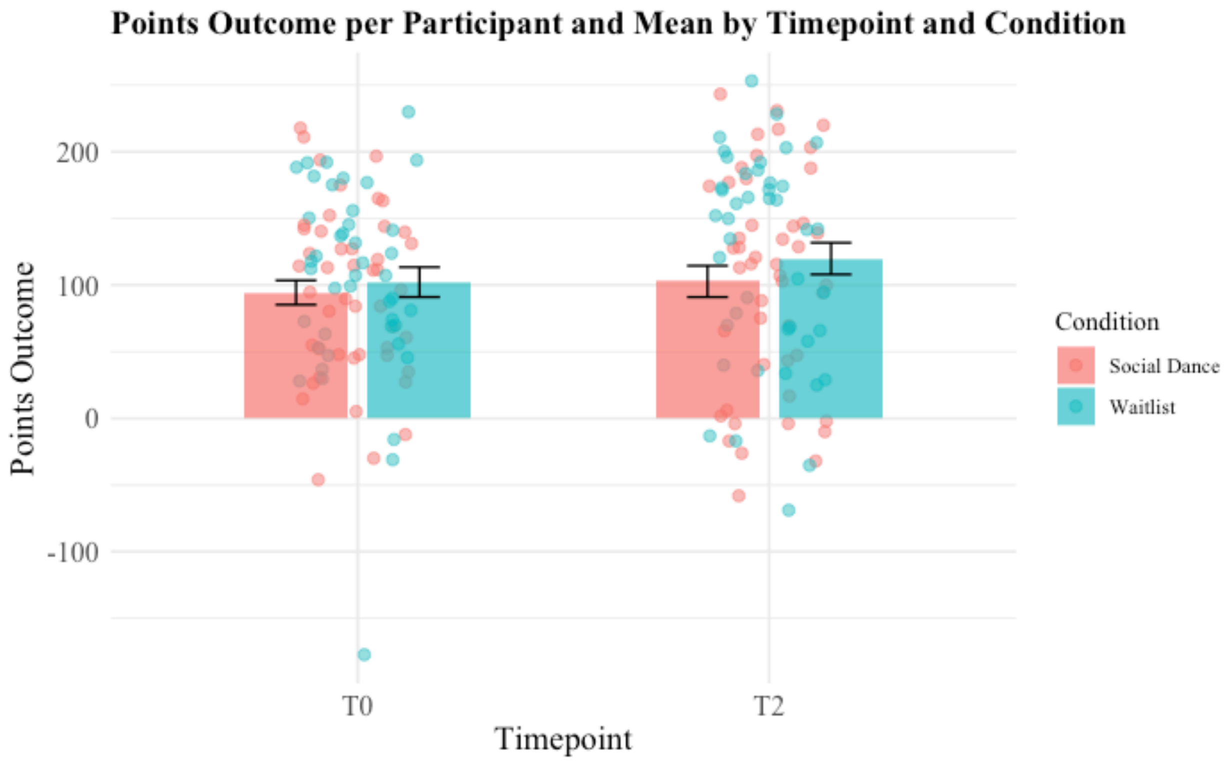

Figure S16. Point Outcome Per Participant and Mean by Timepoint and Condition.

Initial investments

Variable description from methods paper: “participants’ initial investments were averaged from the first five investments they made in the first round with the first five trustees they encountered in the task.” A linear mixed-effects model examining initial investments as a function of timepoint (T0 vs. T2), condition (social dance vs. waitlist), and their interaction, with a random intercept for participant, was conducted. The model revealed no significant main effects (timepoint, b = –0.12, SE = 0.31, t(88) = –0.38, p = .702; condition, b = 0.24, SE = 0.41, t(152.20) = 0.58, p = .564) or interaction of timepoint and condition (b = –0.22, SE = 0.45, t(88) = –0.48, p = .633). These results suggest that initial investments did not significantly differ over time or between experimental conditions.

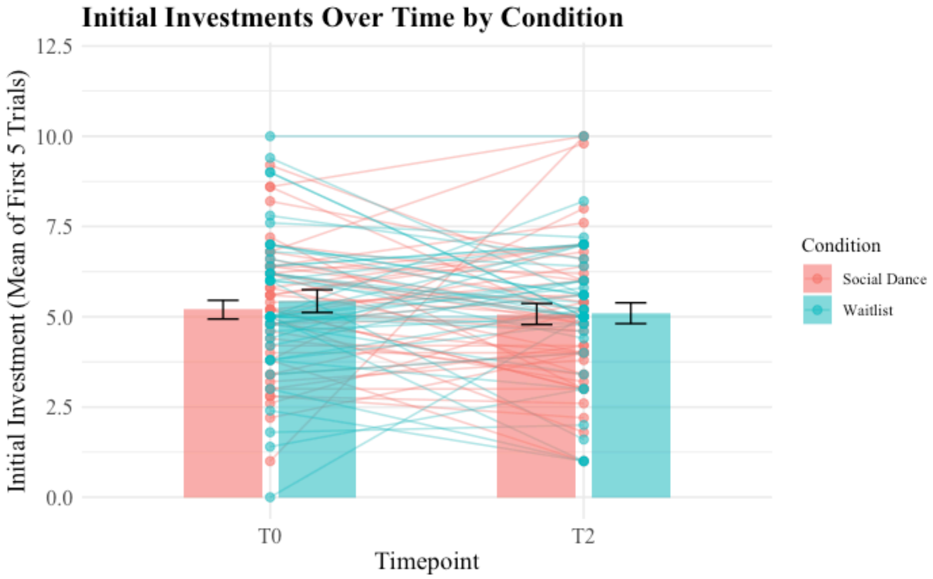

Figure S17. Initial Investments Over Time by Condition.

Investments by trustee generosity condition

Participants' investment in each trustee generosity condition at each time point were summed. A linear mixed-effects model examined the effects of timepoint, condition, and trustee generosity on participants' total investment per trustee generosity condition, with random intercepts for participant. As expected, there was a significant main effect of trustee generosity, $b = 421.36$, $SE = 21.55$, $t(804) = 19.56$, $p < .001$, indicating greater investment as trustee generosity increased. However, no other main effects or interactions were statistically significant, *ps* > .24, indicating that neither timepoint nor experimental condition were associated with differences in investment for each trustee generosity condition.

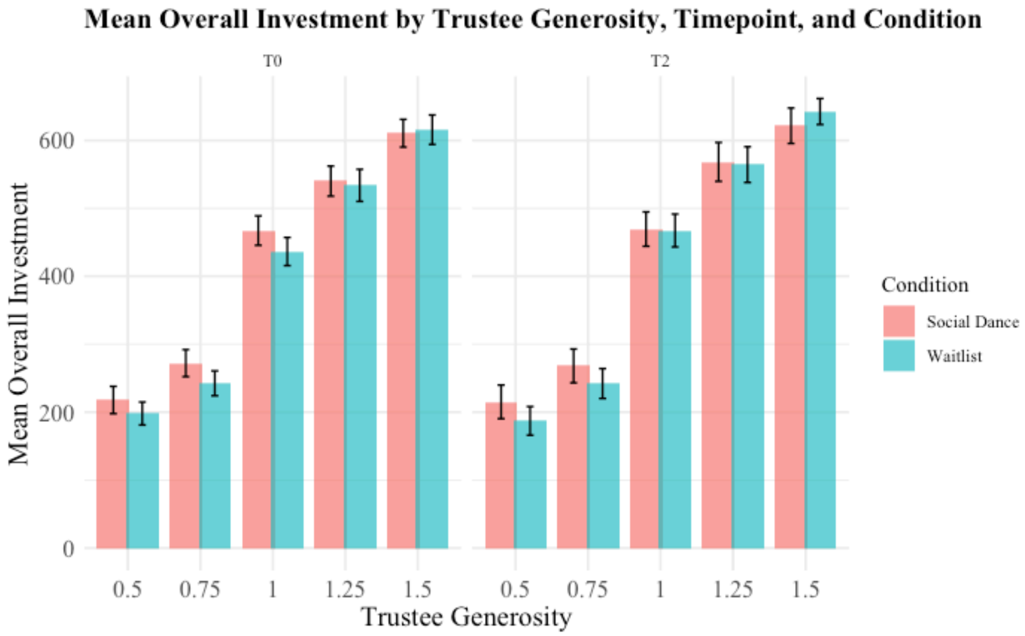

Figure S18. Mean Overall Investment by Trustee Generosity, Timepoint, and Condition.

Change in trustee generosity ratings

At the beginning of each block, participants rated how generous they thought each trustee would be based on just the image of the trustee; directly after completing ten rounds of play with all trustees in the block, participants rated how generous they thought each trustee actually was. These ratings were entered by typing a number between 1 and 100, where 1 indicated "Very ungenerous" and 100 indicated "Very generous." The average change from pre-task generosity ratings to post-task generosity ratings per generosity condition per participant was calculated by subtracting the pre-task generosity rating from the post-task generosity rating per generosity condition per participant.

A linear mixed-effects model was conducted to examine the effects of timepoint (T0 vs T2), trustee generosity condition, and experimental condition (social dance vs waitlist) on participants' average change in generosity ratings. The model included a random intercept for participant to account for repeated measures. While there was the expected significant main effect of trustee generosity condition, $b = 69.42$, $SE = 3.07$, $t(804) = 22.65$, $p < .001$, indicating that generosity ratings varied strongly across trustee generosity levels, the main effect of timepoint was not significant, $b = 4.14$, $SE = 4.60$, $t(804) = 0.90$, $p = .368$, nor was the main effect of condition, $b = 3.52$, $SE = 4.95$, $t(869) = 0.71$, $p = .477$. There were also no significant interactions observed.

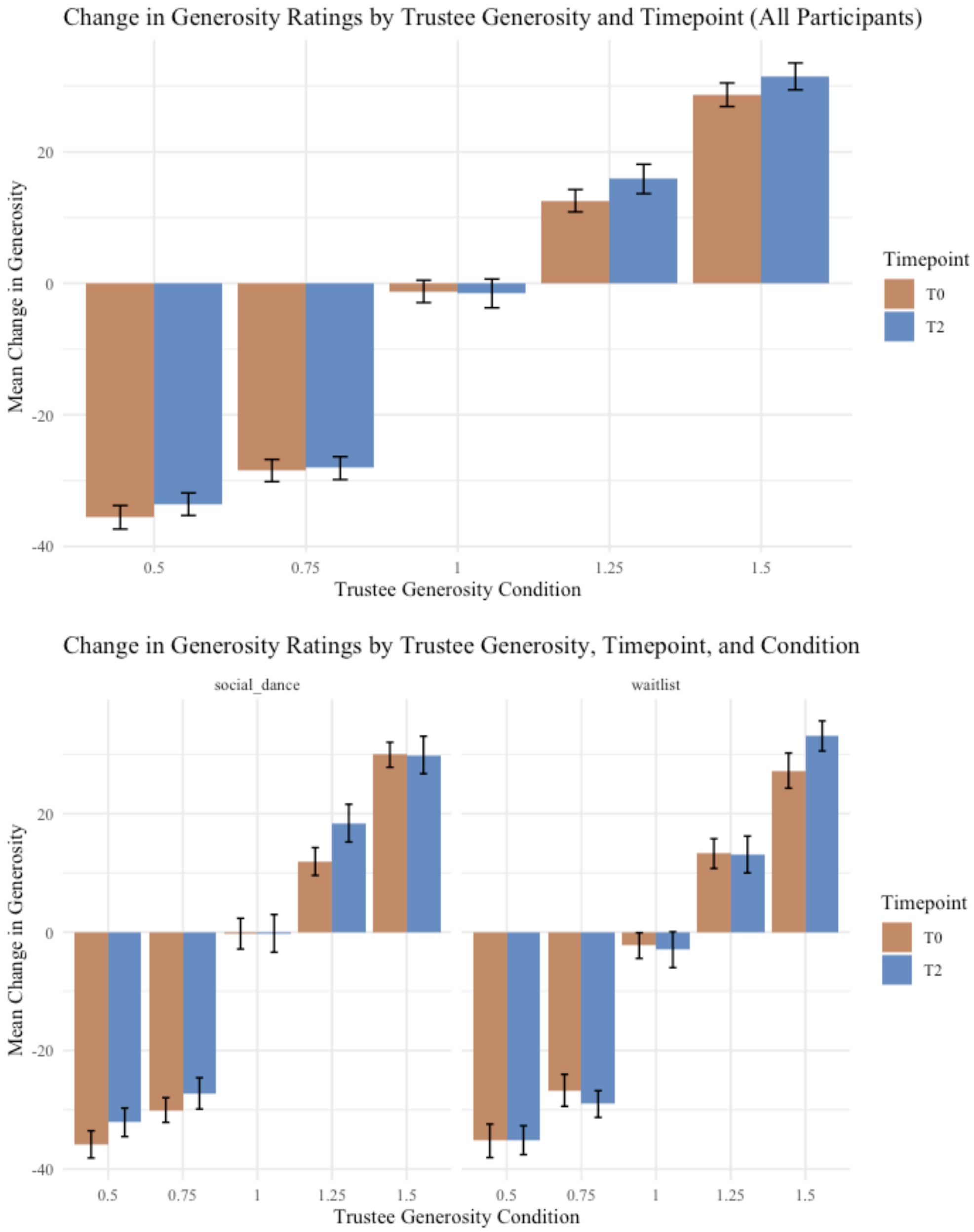

Figure S19. Change in generosity ratings across all timepoints; change in change in generosity ratings by experimental condition.

Change in investment behavior over rounds

To examine whether participants' investment slopes across rounds differed as a function of trustee generosity, condition, and timepoint, a linear mixed-effects model was conducted predicting individual investment slopes from condition (social dance vs. waitlist), trustee generosity condition, and timepoint (T0 vs. T2), including all two- and three-way interactions. Participant-level random intercepts were included to account for repeated observations within participants. The model revealed the expected significant main

effect of trustee generosity condition, indicating that higher trustee generosity was associated with more positive investment slopes across rounds, $B = 0.073$, $SE = 0.013$, $t(804) = 5.61$, $p < .001$. This suggests that participants were more likely to maintain or increase their investments over time when interacting with more generous trustees. A significant main effect of condition was also observed, with participants in the waitlist condition exhibiting significantly more negative investment slopes overall compared to those in the salsa dance condition, $B = -0.064$, $SE = 0.021$, $t(889) = -3.13$, $p = .002$. Additionally, a significant interaction between condition and trustee generosity was found, $B = 0.042$, $SE = 0.019$, $t(804) = 2.26$, $p = .024$. This interaction suggests that the effect of trustee generosity on slope was stronger in the waitlist condition compared to the salsa dance condition. Specifically, while more generous trustees were generally associated with more stable or increasing investments, this effect was greater among participants in the waitlist group. A significant condition by timepoint interaction was also observed, $B = 0.063$, $SE = 0.028$, $t(804) = 2.25$, $p = .024$, indicating that the difference in slope between social dance and waitlist conditions varied from baseline to post-intervention. No other interactions reached statistical significance ($ps > .05$), including the three-way interaction between condition, trustee generosity, and timepoint. Taken together, these results indicate that trustee generosity positively influenced investment behavior over time, and that participants in the salsa dance condition showed more stable investment behavior compared to those in the waitlist condition, particularly at baseline. The waitlist group, however, demonstrated greater sensitivity to trustee generosity in shaping their investment trajectories.

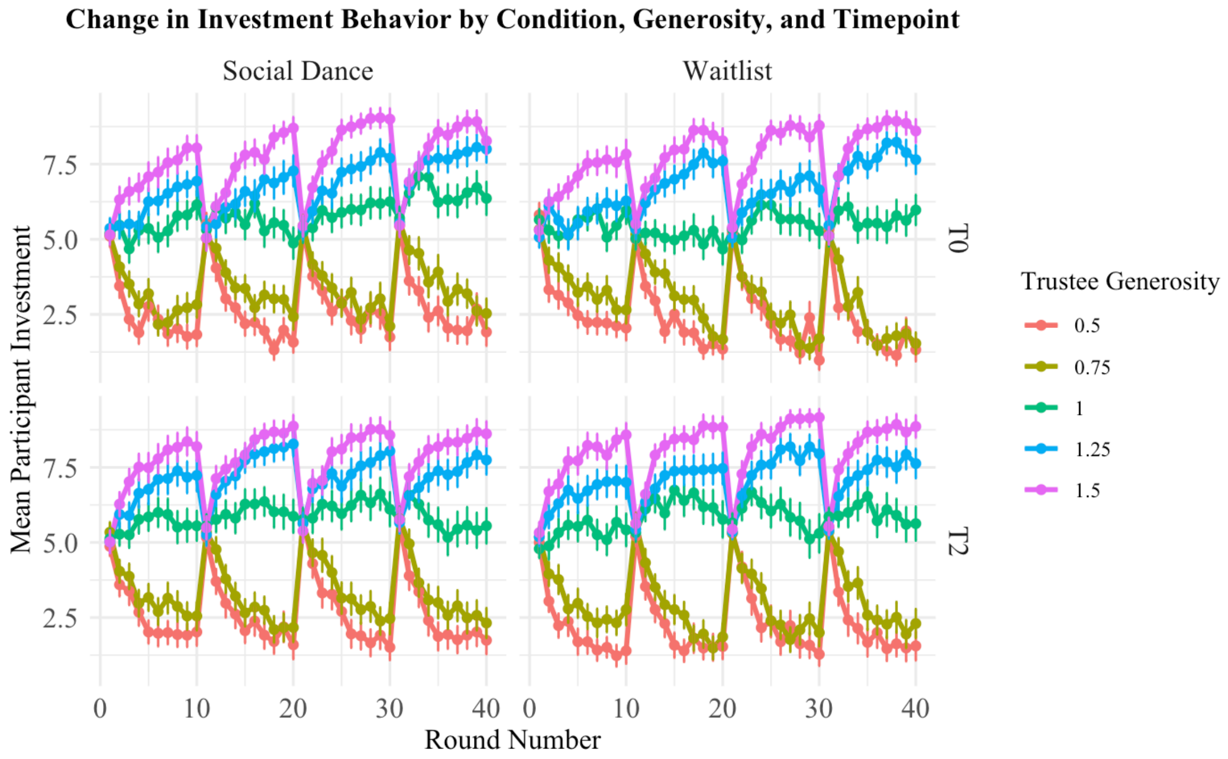

Figure S20. Change in investment behavior by condition, generosity, and timepoint.

Investment plateaus

A linear mixed-effects model examined participants' average investment during the final three rounds (investment plateau) as a function of trustee generosity, timepoint, and experimental condition. There was again the expected significant main effect of trustee generosity, $b = 7.11$, $SE = 0.35$, $t(804) = 20.47$, $p < .001$, namely, participants invested more as trustee generosity increased. However, there were no significant main effects of timepoint or condition, and no significant two-way or three-way interactions among timepoint, condition, and trustee generosity (all *ps* > .19). These results suggest that while investment plateaus increased with trustee generosity, this pattern did not differ by timepoint or experimental condition.

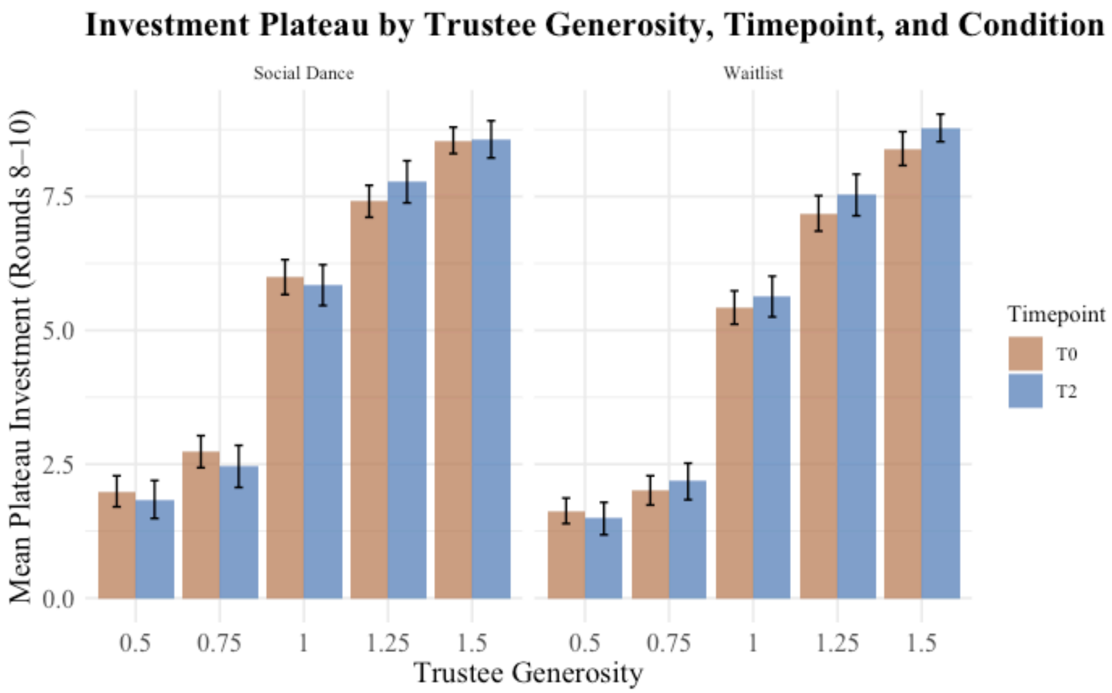

Figure S21. Investment plateaus by condition, generosity, and timepoint.

Desisting from investing

Variable description from task methods paper: "To probe if and when participants ceased to invest at all by trustee generosity condition, especially in cases of unfair trustees, the number of zero investments—in which participants did not invest any points at all with a trustee—per round was calculated for each generosity condition. To be able to compare subgroups with uneven numbers of participants, the percentage of participants *still investing* per trial was calculated as number of non-zero investments divided by the total

number of participant investments on each trial (Figure 4d). Percentages were calculated for each trustee generosity condition and round number, and averaged across blocks."

A generalized linear mixed-effects model was conducted to examine the likelihood of continued investment behavior (coded as 0 = no investment, 1 = any investment) across rounds, as a function of condition (social dance vs. waitlist), trustee generosity, timepoint (T0 vs. T2), and round number (standardized), including all two-, three-, and four-way interactions. The model included random intercepts and slopes for round number by participant to account for within-subject variability across repeated trials. The model revealed the expected significant main effect of trustee generosity, $b = 3.80$, indicating that participants were more likely to invest when interacting with more generous trustees. There were also significant main effects of timepoint, $b = -0.57$, and round number, $b = -0.57$, in such a way as to indicate that participants were less likely to invest at the post-intervention timepoint (T2) and across later rounds of the game. There was a significant condition × trustee generosity interaction, $b = 0.48$, indicating that the effect of trustee generosity on investing behavior was stronger in the waitlist condition compared to the social dance condition. Additionally, a significant condition × round number interaction, $b = -0.58$, suggested that participants in the waitlist condition were more likely to desist from investing across rounds than those in the social dance condition. A significant trustee generosity × round number interaction, $b = 0.65$, indicated that higher trustee generosity attenuated desistance over time. The full four-way interaction among condition, trustee generosity, timepoint, and round number was also significant, $b = -0.34$, suggesting that patterns of desisting across rounds varied by both intervention condition and timepoint, and were further moderated by the generosity level of the trustee. Random effects indicated substantial variability between participants in both overall investment behavior ($SD = 1.57$) and in the rate of change across rounds ($SD = 0.39$), supporting the inclusion of random slopes.

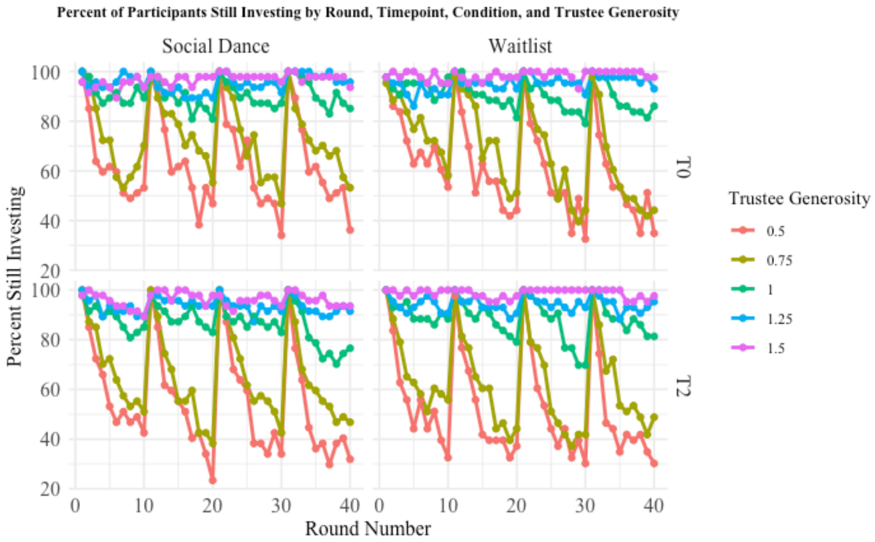

Figure S22. Desisting from investing by condition, generosity, and timepoint.

Appendix G: Additional PHQ-9 Exploratory Analyses

PHQ-9 by Baseline Severity

Baseline PHQ-9 severity has been demonstrated to largely impact endpoint scores (Buckman et al. 2021c). Given the wide range of PHQ-9 scores (0-19) at baseline (T0) in this sample, another LMM was conducted experimentally to examine whether the effect of condition (social dance vs. waitlist) on PHQ-9 scores over time differed based on participants' baseline depression severity (Figure S23). Participants were categorized using clinical PHQ-9 cutoff ranges, comparing those with a score of 10 or above (indicating moderate depression) to those with a score of 9 or below (indicating no depression to mild depression). The model included fixed effects for condition, timepoint (modeled linearly), T0 Category, and their interactions, as well as a random intercept for participants to account for repeated measures.

The model reproduced the previously observed main effect of time ($B = -3.43$, $SE = 0.38$, $t(364) = -9.04$, $p < .001$) but not condition ($B = 1.15$, $SE = 0.64$, $p = .078$), and condition by time interaction ($B = 1.56$, $SE = 0.55$, $t(364) = 2.82$, $p = .005$), suggesting that social dance participants had significantly lower endpoint PHQ-9 scores compared to those in the waitlist group even after adjusting for their baseline PHQ-9 scores.

Additionally, the model revealed a significant time point × T0 Category interaction ($B = 2.96$, $SE = 0.75$, $t(364) = 3.96$, $p < .001$), indicating that baseline depression severity influenced symptom change over time, and the main effect of T0 Category was also significant ($B = -3.82$, $p < .001$), indicating that participants with lower baseline symptoms consistently reported lower PHQ-9 scores throughout the study.

Despite this, the three-way interaction between condition, time point, and T0 Category was not significant ($p = .160$), indicating that the effect of the social dance intervention did not significantly differ by baseline depression severity. In other words, while baseline PHQ-9 scores were associated with the amount of overall improvement, participation in the social dance intervention was associated with greater symptom improvement regardless of baseline symptom severity (Figure S23).

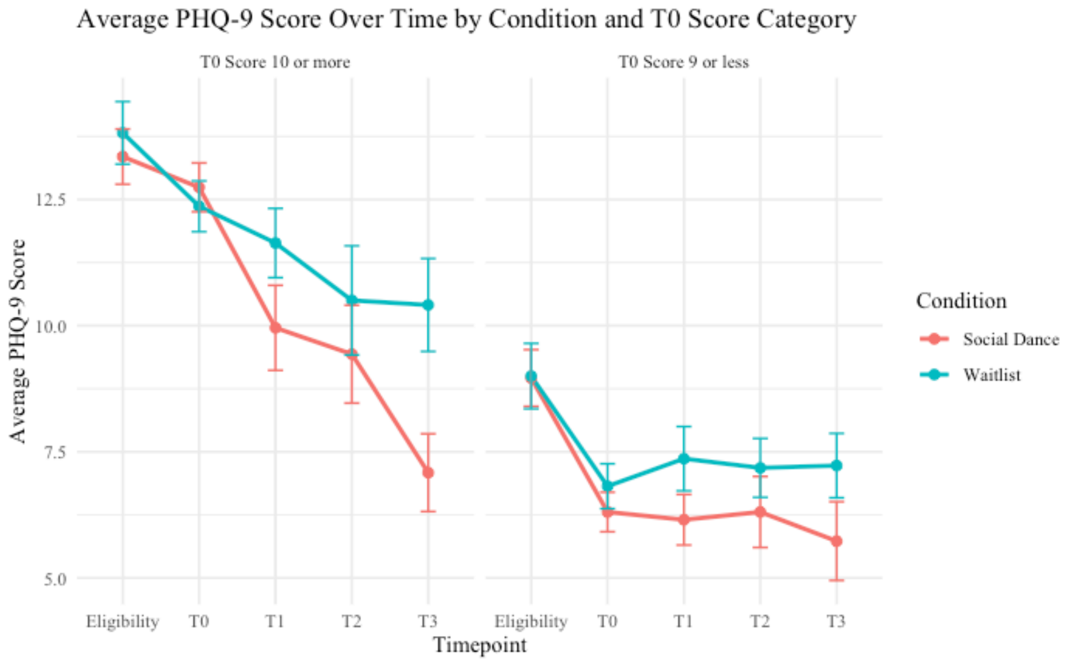

Figure S23. Average PHQ-9 Score Over Time by Condition (Social Dance vs. Waitlist) and PHQ-9 T0 Category (Moderate, PHQ-9 Score of 10 or above, vs. None to Mild, PHQ-9 Score of 9 or below) for all participants (*n = 93*). The social dance group had 26 participants (53%) with a T0 score of 9 or less, and 23 participants (47%) with a T0 score of 10 or above, while the waitlist condition had exactly 22 participants (50%) in each T0 category.

Appendix H: Qualitative and Quantitative Acceptability Measures

Due to the structure of the study, control group participants were offered the opportunity to participate in salsa classes either as part of the study or as not part of the study after completing T3; none of the control group participants both chose to complete the salsa classes as part of the study and also finished all of the time points. Therefore, acceptability of intervention (e.g., salsa classes) data is only from the experimental group. Due to a glitch in the questionnaire system, three participants completed the acceptability measure twice; therefore, responses of three participants are represented twice in the acceptability data. Participants responded to 10 Likert-scale acceptability of intervention questions, as well as two free-response questions. Some participants also provided free-response feedback that was included with permission with the rest of the free responses.

- **Did you like the salsa classes in this study?**

  Participants responded to this question by choosing between the following responses (single-selection buttons): Strongly dislike / Dislike / No opinion / Like / Strongly like. Below is a histogram of the frequency of their responses:

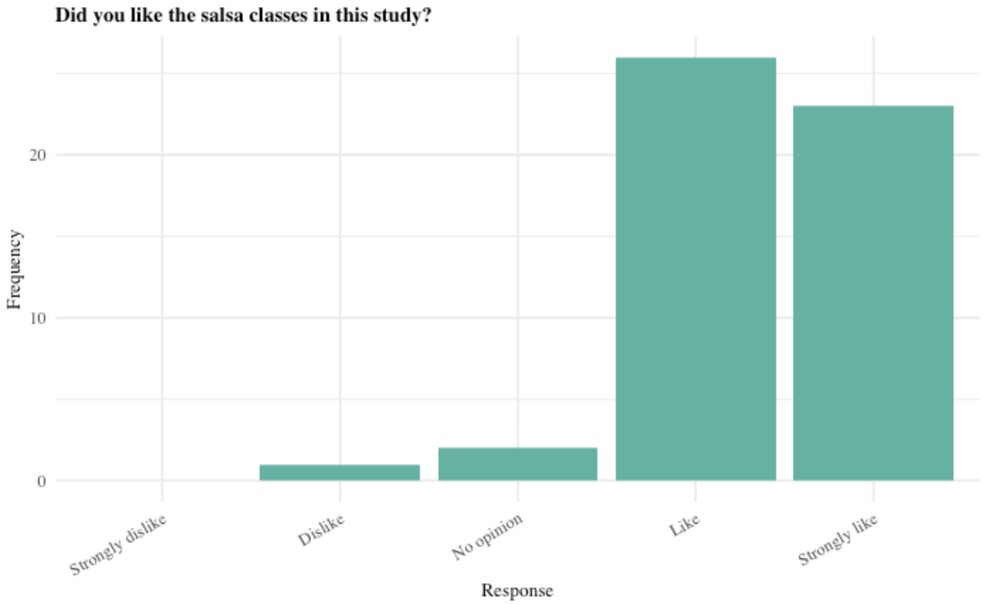

- **How comfortable did you feel engaging in the salsa classes in this study?**

Participants responded to this question by choosing between the following responses (single-selection buttons): Very uncomfortable / Uncomfortable / No opinion / Comfortable / Very comfortable. Below is a histogram of the frequency of their responses:

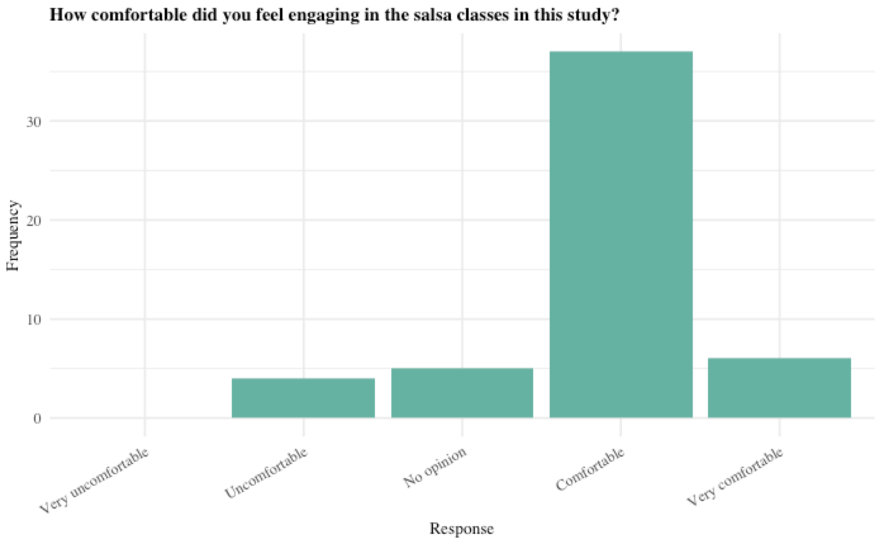

- **How much effort did it take to participate in this study?**

Participants responded to this question by choosing between the following responses (single-selection buttons): No effort at all / A little effort / No opinion / A lot of effort / Huge effort. Below is a histogram of the frequency of their responses:

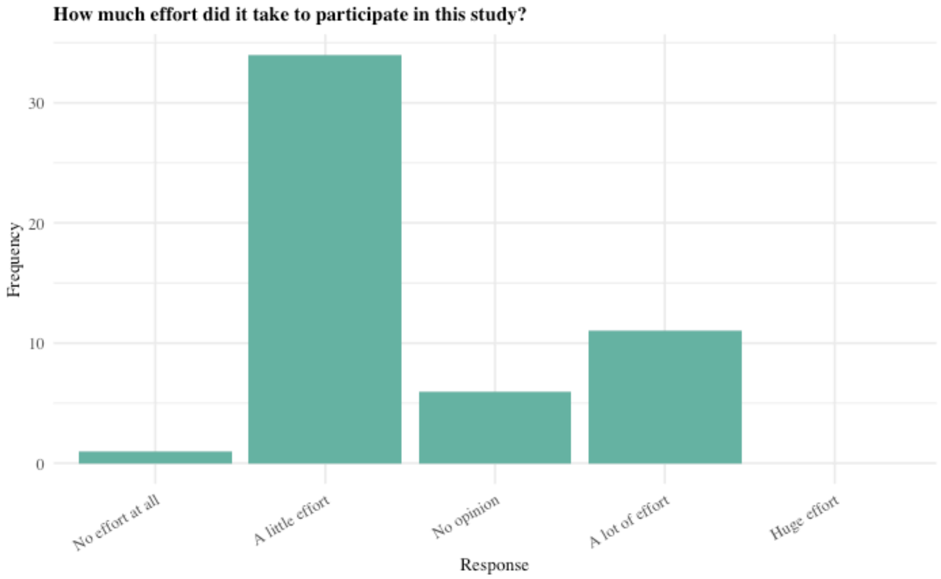

- **How well did you feel that these salsa classes were a good fit for young people with low mood?**

Participants responded to this question by choosing between the following responses (single-selection buttons): Very poor fit / Poor fit / No opinion / Good fit / Very good fit. Below is a histogram of the frequency of their responses:

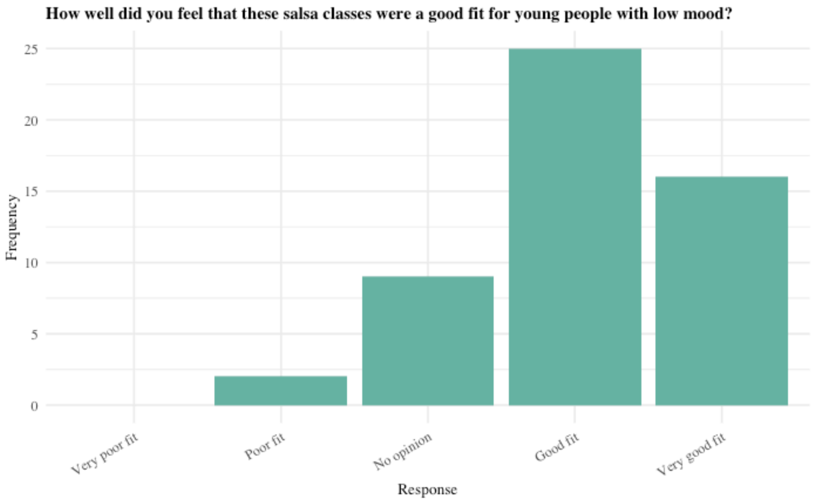

- **The salsa classes have improved my feelings of low mood; I feel my mental health has improved for taking the salsa classes:**

Participants responded to this question by choosing between the following responses (single-selection buttons): Strongly disagree / Disagree / No opinion / Agree / Strongly agree. Below is a histogram of the frequency of their responses:

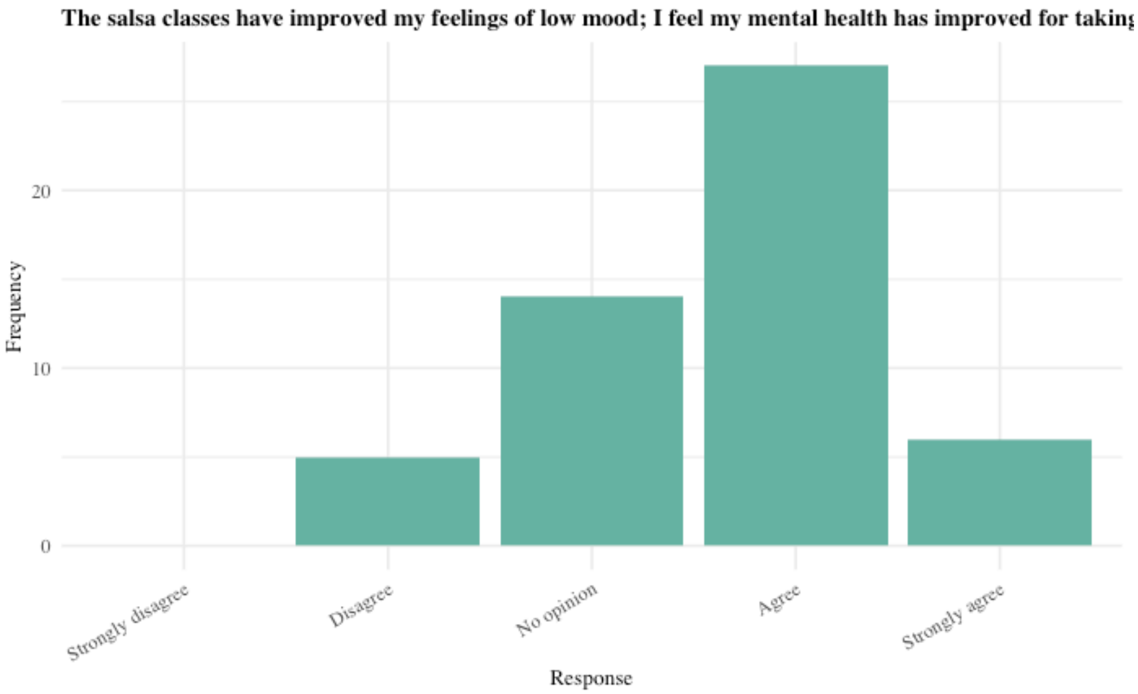

- **It is clear to me how social movement (salsa) classes might help improve my mood:**

  Participants responded to this question by choosing between the following responses (single-selection buttons): Strongly disagree / Disagree / No opinion / Agree / Strongly agree. Below is a histogram of the frequency of their responses:

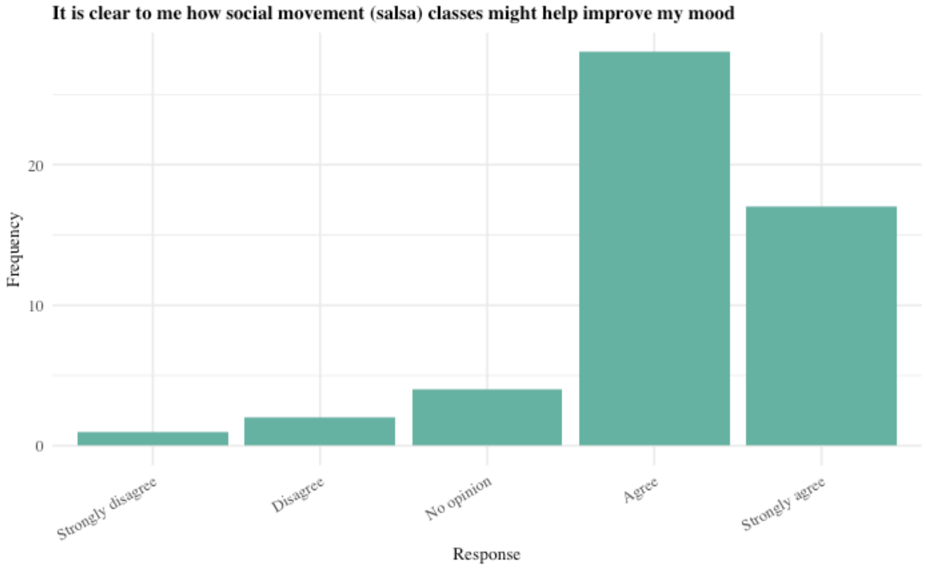

- **How confident did you feel about your ability to participate in the salsa classes?**

  Participants responded to this question by choosing between the following responses (single-selection buttons): Very unconfident / Unconfident / No opinion / Confident / Very confident. Below is a histogram of the frequency of their responses:

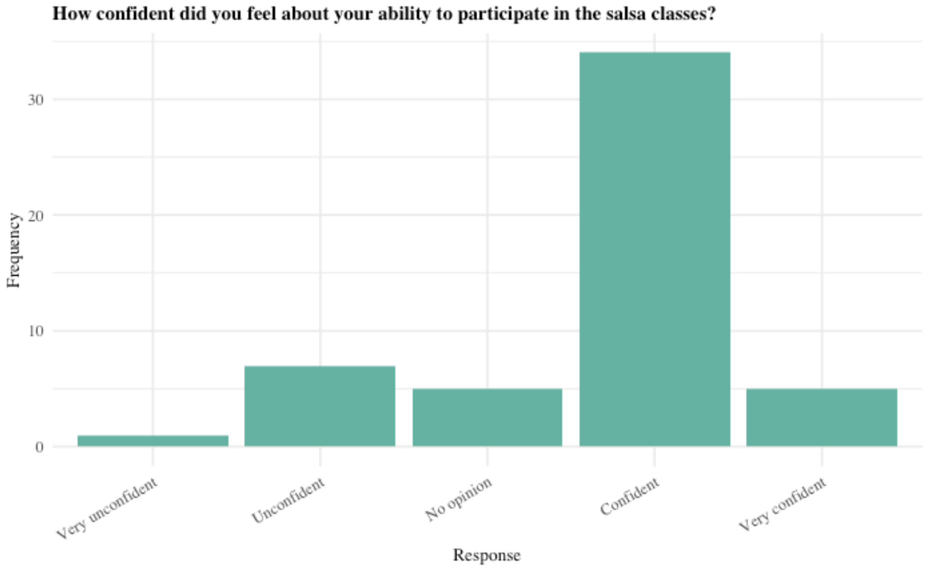

- **Engaging in the salsa classes interfered with my other priorities:**

Participants responded to this question by choosing between the following responses (single-selection buttons): Strongly disagree / Disagree / No opinion / Agree / Strongly agree. Below is a histogram of the frequency of their responses:

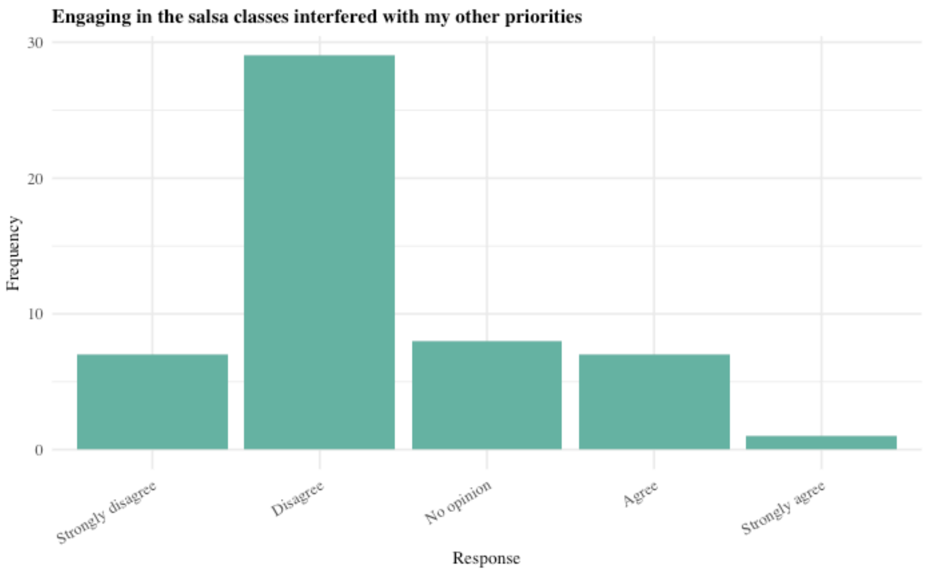

- **How acceptable were the salsa classes as part of this research to you?**

Participants responded to this question by choosing between the following responses (single-selection buttons): Completely unacceptable / Unacceptable / No opinion / Acceptable / Completely acceptable. Below is a histogram of the frequency of their responses:

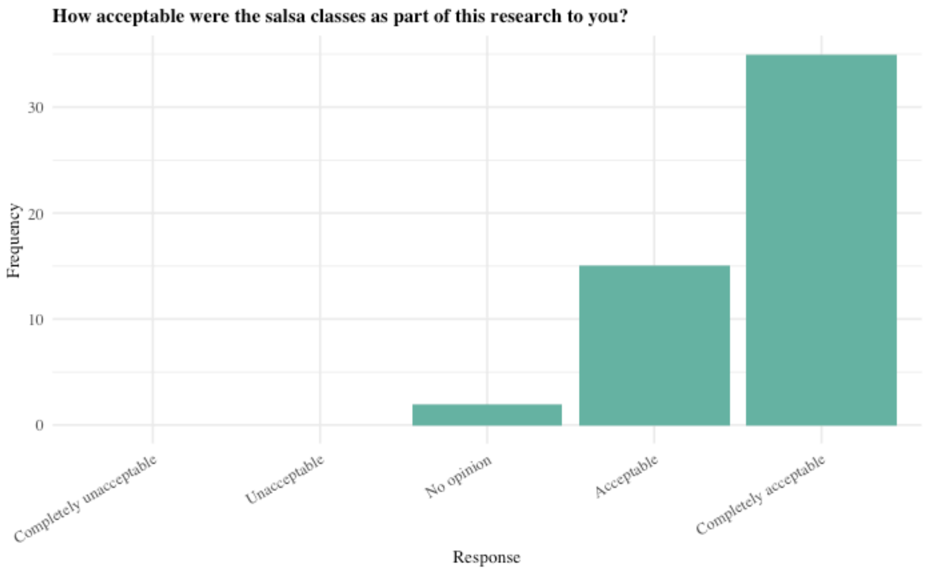

- **How likely would you be to recommend this study / social dancing to a friend?**

Participants responded to this question by choosing between the following responses (single-selection buttons): Very unlikely / Unlikely / A bit unlikely / No opinion / A bit likely / Likely / Very likely. Below is a histogram of the frequency of their responses:

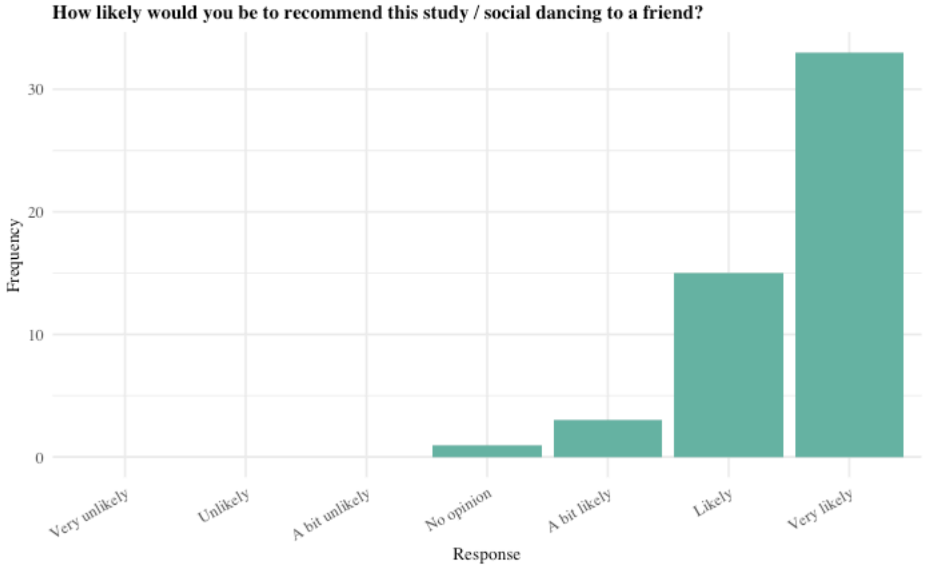

3.6.2. Free Response Items

Please feel free to provide any other comments, thoughts, or feedback on these questions or this study:

1	I loved the sessions, Brennan and the other teachers were super supportive and helpful and fun !
2	Just wanted to reiterate how much I have enjoyed participating in the study! Not only did it allow me to dis cover a new hobby (one that I have been wanting to do for ages!), it was something that I was also able to s hare with my friends who also participated in the study. I think I found getting the motivation to get up and go to classes difficult, but I always felt energised + in high spirits after the classes! It was a really friendly, welcoming environment designed for beginners and I think that contributed to people feeling more at ease a s the classes progressed.
3	I really loved the classes and made some friends from going! While I am not 100% sure if they improved m y mental health long-term I always felt better after taking a class than I did before!
4	Felt anxious switching partners all the time, especially when some people were clearly of a higher dance level than others (eg. dance experience but not necessarily salsa) Found it confusing to switch from leader to follower every few minutes. Made me feel embarrassed quite frequently. Would've prefered to stay doing one part and felt i could have progressed more. I did feel salsa improved my mood when i clicked with someone and we were getting it right, but quite frequently i'd get difficult partners. eg. one partner trying to teach me herself rather than following the class direction and insisting she follow me with her eyes closed to help me learn, even after i told her i was uncomfortable doing so.

5	Undeniably immediately after the salsa classes my mood improved massively, everytime. I would say defin itely extending into the next day as well. I think overall, there are so many factors that determine my mood that it would be hard to say that the classes improved my mood on the whole of the whole term; however th is doesnt take away from the fact that after every salsa class my mood was greatly improved. I hope that ma kes sense!
6	I think it would be beneficial to have the option to provide context to our questionnaire answers with what else is going on in our lives. I strongly believe that the salsa classes had nothing but a positive impact upon my mental health, but I'm not sure how accurately my responses reflect that. For example, a 12-week Oxford term slowly grinds me down as well as getting through the anniversary of my mother's suicide and whilst salsa classes are a moment of respite during the week I think it's unrealistic to expect that one hour to be able to counteract the shitstorm of rubbish I'm trying to juggle. Equally, after Christmas I go on a yoga retreat in Sri Lanka and my mental health is miraculously healed and I'm having 10/10 days for the first time in months/years because of a drastic change in environment.
7	Connections with people were very brief and cannot build trust though dancing was very enjoyable
8	The instructors were very friendly and welcoming, I think that helped a lot!
9	It was fun! Always enjoyed after salsa with no regret for doing it - doing it next term as a result
10	Just wanted to thank for this opportunity, it truly was a really fun and wonderful experience. Having had fe w dance classes before, I didn't think I would enjoy this as much as I did. So thank you!
11	Really enjoyable. Switching up the people from the classes could be interesting? maybe hard to implement
12	It was a very well run study, extremely easy to take part in and very streamlined. The salsa teachers were al so incredibly kind and funny and made the experience truly enjoyable
13	I really enjoyed the lessons
14	I really enjoyed the salsa and the survey each week after salsa wasn't too difficult.
15	Loved the classes!
16	I had never done salsa before but really enjoyed it :)
17	the teachers enthusiasm really helped with learning the dance and improving everyones moves.
18	I have been having an extremely difficult time this summer and ended up being put on antidepressants due t o my extreme low mood. These salsa classes were sometimes the only thing that would get me out of my b ed. I always felt at least a little bit better after them. I also really enjoyed the daily "how are you feeling" te xts because it forced me to take a moment and check in with myself.
19	i really enjoyed the salsa classes, especially when it was a smaller class
20	Loved the study very much! It was a great way to stay accountable to taking weekly salsa classes.
21	I think the salsa classes could be a productive method for imporving the mood and mental health of young people. This is subject to the individual of course. Although it being key, I found engaging with lots of new people challenging as I don't like phsyical touch very much - I hadn't thought abut it prior to the class. Des pite this, the actual learning of the dance was fun and I think it will be a nice way for me to socialise with p eople in the future. I found it hard to keep up to date with the daily mood measure too which I am afraid ma y have let down my results. Perhaps a weekly message would help for people to keep up and give a more g eneral review - I understand this would reduce you data set considerably though, and may not provide an ac curate represnation of people's true moods. Thank you though, so much for teaching me and having patien ce. I think your teaching method is really effetive because you're senstive and genuinely listen to people's st ruggles. I hope this project has been successful for you - all the best for the future!!
22	I am very greatful to [the<br>instructor] for being so accesible, quick to reply to queries and positive throughout the study.

23	I think for someone with very low mood who is also an introvert the clases could be quite exhausting becau se everyone is very positive. I would say I am an extrovert so I enjoyed it but there are people I know who I think would not. Perhaps it is also dependant on the participant's personality type.
24	Really enjoyable - I want to keep attending!
25	I wasn't too sure about the meaning behind the facial reaction recognition online tasks. At times they got qu ite repetitive.
26	There was a noticeable change in my mood after taking each class, and I felt happier and more able to appr oach other tasks in my week. I really noticed the absence of the classes while at home over Christmas.
27	I do think it's important to clarify that one of the reasons why I think these classes were so beneficial to me was because I was able to coerce a friend into doing the study with me. While most of the class were stran gers to myself, having a familiar face in the group (even if we didn't dance together every class) made this a very easy and quick transition. I do think that if I'd done this completely on my own that I would have eve ntually grown to like it as much as I do now but it would have most likely taken a while longer for me to ha ve that level of comfort and I'm not sure I would have reached that point by the 8th class.
28	Being new to the country, my mood was affected a lot by the changes in my life. Going for salsa gave me s omething to do and break my routine during the week. I had fun and found a lot of people whom I felt I cou ld bond with given the chance. I really appreciated how Brennan treated me personally. She was always so bubbly and welcoming; she complimented my dancing whenever we got to be salsa partners. This was grea t and now I have a precious memory for the books.
29	Certain life events occured whilst I was participating in the study which I feel probably affected my mood b eyond the scope of its research, particularly with regards to the daily mood questions. However, even when I felt little motivation to go to the lessons I was always glad I went to them and left feeling more energetic.
30	I found it difficult to rate my mood numerically but I can't necessarily think of a better way to do this
31	I know this may interfere with the study's goals, but I didn't really find out if there would be a broader salsa community to get involved in? Or beginner-level social dancing.
32	Some of the questions in the questionaire might have strong orientation towards the research goal. I am not sure if I have provided the most honest views in answering them. I suggest change the wording of some of t he questions next time.
33	great fun!
34	Absolutely loved the classes, they have shown me a new hobby, taught me how to listen to my own and oth ers bodies, and the musical, expressionate and social aspects of the class are literally perfect for low mood.
35	i very much enjoyed the salsa as it was a low-pressure, comfortable envinroment to socialise
36	It was a great time taking the class! Interested in seeing the result!
37	I know you have a lot of participants so it would be a lot of effort but maybe for a future iteration: i think in terviews with participants could be quite valuable - I'm involved in a similar project where people get free music classes and do some mood questionnaires etc and also interviews, and lots of participants I've spoken to gave really rich responses in interviews that gave a totally different impression than their responses to th e questionnaires

38	I enjoyed the salsa classes, however, it would have been more effective to be dancing with the same people week after week rather than constantly being reintroduced to strangers and then not seeing people with who m you may have established rapport or connections in the following weeks. I also think there should have b een more opportunities for one-on-<br>one teaching to ensure that people who don't feel confident in their abilities don't feel even worse about the mselves if they aren't getting the steps, i.e., more teachers at each salsa class. I don't know how confoundin g variables were considered for this study. I.e., how do you accurately measure if salsa was the thing that le d to a change in mental state and not something else? For example, maybe a spiritual/emotional breakthrou gh occurred during the timeframe of this study, or an enriching vacation, new relationship, change in life ci rcumstances etc
39	everything was good well structured and thoght through

Additional feedback provided through direct messages, reported with permission:

40	I had an amazing time. Thank you so much for giving me more joy in my life and a new hobby that allows me to express myself. You've genuinely helped me and my life by doing this study.
41	Honestly I've loved doing the classes and am planning to come back a bit this term. I genuinely did always feel better after them and me and my partner taught some of our friends some of the basics!
42	we really enjoy it! Some of us are housemates, and we practice together at home all the time. We definitely wouldn't have tried salsa otherwise, but are definitely planning to continue!
43	I'm going to miss the daily mood texts! What a pleasant notification to get on my phone - most notifications aren't so pleasant
44	recently we (some of the people who did the salsa class together) went out for drinks together!
45	I started salsa as part of one of your studies last year and I just wanted to quickly say thank you so much!! It helped me manage my mental health so much more healthily in my final year of uni alongside my meds. I went from being unsure if I would drop out because I felt so rough and was struggling so much, to getting the best marks of my degree :) I've been dancing ever since and absolutely loving it (RIP my bank account though haha). I am currently living in an area with no dance classes nearby and have definitely noticed changes in my mood (idk if that's of interest). I'm more than happy to be contacted about any other similar research you do in the future!

Themes:

- Characteristics of teachers helped, broadly defined (e.g., enthusiasm, friendly, welcoming)
- Experience level of people in the class mattered
- Improvements in mood after classes, for variable lengths of time, and motivation to go to classes
- Being able to build trust with / have consistent people in classes is preferable; discomfort with switching partners more frequently / if not feeling confident
- Other factors influence mental health (e.g., Oxford term, positive and negative external life events) beyond salsa, and participants made a point to say that they weren't sure that the mental health measures accurately reflected this / captured the effect of salsa in light of everything else going on

Is there anything we could have done to improve the experience of taking part in this study? (non-N/A responses reported:)

1	It was quite challenging to learn to both lead and follow which would cause me to lose confidence a bit. Per haps allowing people the option of whether they would learn one or the other would help.
2	Not really - I think it was really good how the number / day of sessions was quite flexible so there wasn't to o much pressure to go if you weren't in the mood. Honestly, a really enjoyable study which felt like it had t angible benefits in terms of mental AND physical health which was really nice.
3	Very difficult to slot around everyone's availability but in terms of the actual salsa, it would have also been fun to stay in fixed classes to get to know people better/all learn at the same pace :)
4	Maybe have a different salsa teacher to the person running the study so it felt more annoymous / research c onfidentiality ? Ask how has your day been, rather than how do you feel in this moment in the daily texts, because i generally only check my phone and reply to things in a down moment
5	I would shorten some of the tests that were required.
6	No, you've been great :)
7	People will not be likely to engage in the class if they are in a really low mood and not (sexually) confident at all. Though when you're dancing, you dance with the flow. And the biases are only the ones who want t o dance will participate in the study. Hope this helps. Best wishes on the study.
8	It might be helpful do describe mood in words rather than on a numerical scale - sometimes I wasn't quite s ure how to interpret my mood on a 10 point scale.
9	Nothing that I can think of.
10	Maybe for people already experiencing intense anxiety or low mood it might be hard to engage in so much small talk in such quick succession and it might help for the initial classes to focus just on the dancing and ease into the social aspect later to make it less daunting for those people
11	sometimes there were too many people in the room
12	I liked the study bt I eould have appreciated being sent the study link every week and I didn't enjoy the dail y texts as they came at irregular times and I wasn't aware that would be a part of the trial.
13	N/A was all good
14	Not really. Perhaps smaller groups?
15	Having to go to 5 or 6 in an 8 week term was a bit tricky to fit in, would have been easier over 2 terms as te rms are so short.
16	smaller classes, more time to get comfortable with dancing partner. I found it harder to learn the moves wh en constantly switching, when we just learned how it works with one partner, another does things slightly d ifferently so it was like learning the move all over again. sticking with the same partners allows the comfort ability to build, therefore the strength of the moves as well.
17	I think it was really good on all aspects.
18	split by ability maybe? sometimes we went through moves too slowly.
19	no
20	Everything I've thought of is above
21	A very technical thing, but there were a couple of times where I wasn't able to log attendance and rate my mood online immediately after the class through the QR code as I didn't have my phone/ my phone died, so I wondered if it might be worth offering an paper-<br>alternative or 'go to this website later!' option in these cases to make sure attendance is still recorded.

22	Unsure
23	N/A [the instructor] was so nice and helpful !
24	Perhaps making it easier to attend the other slots during the week.
25	I think that everything has been extremely accessible on my end so I don't have anything in mind for impro ving the experience.
26	Possibly explain at the start that it's best to go to one of the classes consistently rather than switching aroun d (as in, go to all the monday classes rather than flitting between monday, tuesday, wednesday etc), since th is will help with keeping consistency in terms of what you're learning.
27	I like salsa dancing classes, but sometimes i might feel uncomfortable dancing with boys. It's easier for me to dance with girls.
28	maybe some people can be assigned with fixed dance partners? just in case they feel uncomfortable dancin g with strangers
29	To imform the content of the questionnaires and the time needed to complete them when recruiting the part icipants.
30	more salsa lessons, and more options to continue salsa outside of the classes
31	no, i had a good time
32	Was great! N/A
33	Made the salsa classes more organized and structured. However, they were free, so I think it was a great op portunity to have regardless.
34	not really,, it was easy

Appendix I: Study Recruitment Flyer

Aged 18-24, and feeling a bit low in mood?

You may be eligible for a research study.

We are looking for volunteers to participate in up to eight free salsa dance classes and to respond to questionnaires and online tasks pertaining to your mood, feelings, and other demographic factors.

You will be compensated for your time.

No dance experience expected or required!

Interested in learning more?

soma.study@psych.ox.ac.uk

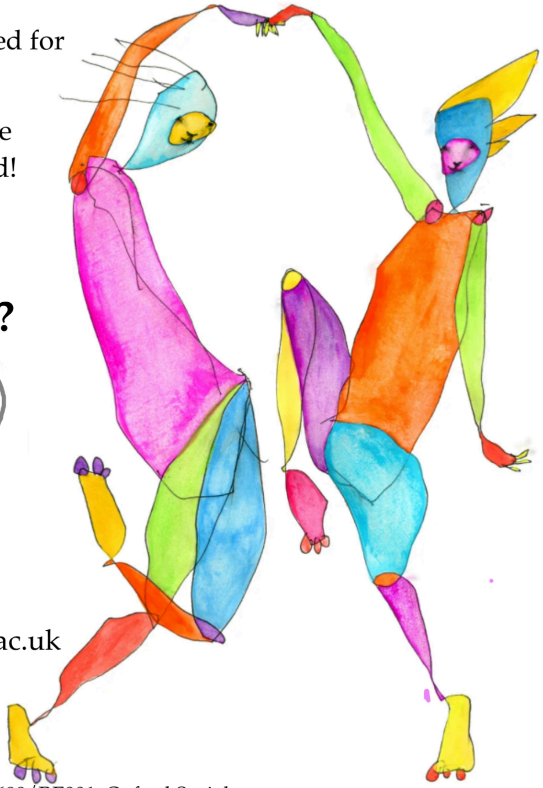

Ethical Approval Reference: R85689/RE001; Oxford Social Movement Activation (SOMA) Study

Art by Brian Andreas; used with direct permission
